# Evaluation of *Zamia floridana* A. DC. Leaves and Its Isolated Secondary Metabolites as Natural Anti-Toxoplasma and Anti-Cancer Agents Using In Vitro and In Silico Studies

**DOI:** 10.3390/metabo13010010

**Published:** 2022-12-21

**Authors:** Hosam M. El-Seadawy, Kamilia A. Abo El-Seoud, Mona El-Aasr, Haytham O. Tawfik, Wagdy M. Eldehna, Amany E. Ragab

**Affiliations:** 1 Department of Pharmacognosy, Faculty of Pharmacy, Tanta University, Tanta 31527, Egypt; 2 Department of Pharmaceutical Chemistry, Faculty of Pharmacy, Tanta University, Tanta 31527, Egypt; 3 Department of Pharmaceutical Chemistry, Faculty of Pharmacy, Kafrelsheikh University, Kafrelsheikh 33516, Egypt; 4 School of Biotechnology, Badr University in Cairo, Badr City 11829, Egypt

**Keywords:** *Zamia floridana*, toxoplasmosis, cytotoxicity, in silico docking, bioflavonoids, phenolic acid derivatives

## Abstract

Toxoplasmosis and cancer are life-threatening diseases with worldwide distribution. However, currently used chemosynthetic treatments are not devoid of their own intrinsic problems. Natural metabolites are gaining attention due to their lower side effects. In this study, we investigated for the first time *Zamia floridana* leaves extract and its different fractions for their toxoplasmocidal activity, using *Virulent RH Toxoplasma gondii*, and cytotoxic activity against MCF-7 and HCT-116 cancer cell lines using MTT assay. The *n*-butanol fraction was the most potent fraction against *T. gondii* with an EC_50_ of 7.16 ± 0.4 µg/mL compared to cotrimoxazole (4.18 ± 0.3 µg/mL). In addition, the *n*-BuOH fraction showed a significant cytotoxicity against MCF-7 and HCT-116 with IC_50_ of 12.33 ± 1.1 and 17.88 ± 1.4 µg/mL, respectively, compared to doxorubicin (4.17 ± 0.2 and 5.23 ± 0.3 µg/mL, respectively), with higher safety index against normal cell line (WISH). Therefore, the *n*-BuOH fraction was investigated for its phytochemicals using extensive chromatographic techniques, which led to the isolation of six compounds that were fully characterized using different spectroscopic techniques. Three biflavonoids (**1**, **2** and **4**) in addition to two phenolic acid derivatives (**3** and **5**) and a flavonoid glycoside (**6**) were isolated. Compounds (**1**, **3**, **5** and **6**) were reported for the first time from *Z. floridana*. In silico docking studies for toxoplasmocidal and cytotoxic effects of these compounds revealed that compounds (**1**, **2**, **4** and **6**) have promising inhibition potential of either thymidylate synthase-dihydrofolate reductase (TS-DHFR) or cyclin dependent kinase 2 (CDK2) target proteins. This study is considered the first report of chemical and biological investigation of *Z. floridana* leaves.

## 1. Introduction

Gymnospermous plants have been documented since 300 BC [1]. The order of cycadales is one of the largest groups of living gymnosperms. It is commonly referred to as the cycads. *Cycadaceae* and *Zamiaceae* are the most important families in this order due to their large number of species and wide range of biological activities such as *Cycas revoluta*, that has cytotoxic and antioxidant activities and *Cycas rumphii*, which was previously reported as a natural source for toxoplasmocidal and cytotoxic agents [2,3]. In addition, various biological effects have been reported for different species of *Zamiaceae* such as the antimicrobial effect of *Dioon spinulosum* and the antileishmanial activity of *Zamai lindenii* [4,5]. The genus *Zamia* was found to exert a wide range of significant biological effects due to their high content of biflavonoids, flavonoids, lignans, phenolic acids, fatty acids, sterols and amino acids [6,7,8,9]. *Zamia floridana* A. DC. is one of many *Zamia* species that belong to the family *Zamiaceae*. It is a dioceous small evergreen shrub. It has another synonym as *Z. integrifolia*, which refers to the entire leaflet edge and its name was derived from the Latin words “integer”, which means “entire” and “folium”, which means “leaf”. *Z. floridana* has subterranean tuberous stems with linear to lanceolate glabrous leaflets with blunt apices and entire margins. Its occurrence ranges from the extreme southeastern Georgia state of the USA to southward through Florida [10]. *Z. floridana* has a widespread use as a food among Florida Indian people by preparing a flour base from the roots after washing or boiling to remove the toxins [11]. There are no previous studies about the phytochemicals and the biological effects of *Z. floridana*.

*Toxoplasma gondii* is one of the major parasites affecting human health and animal productivity since it infects humans and nearly all warm-blooded animals [12]. It causes toxoplasmosis, which is a widespread zoonotic disease affecting about one-third of people worldwide [12]. In immune-compromised individuals, *T. gondii* can cause pneumonia, encephalitis or other dangerous diseases. It can also result in serious congenital defects in children born to infected mothers [13].

The concurrent treatment of toxoplasmosis includes the use of two drugs (sulfadiazine and pyrimethamine). These medications work by inhibiting the folate metabolism, which consequently prevents the production of DNA and, ultimately, the replication of tachyzoites. However, the use of these current chemotherapeutic drugs is little limited due to their side effects [14].

Cancer is the second most common cause of mortality. One in eight deaths globally is due to cancer. The toxicity of chemotherapeutic drugs that are routinely used for cancer treatment sometimes creates significant side effects [15]. Consequently, there is an urgent need to find effective and safe agents with lower toxicity. 

The previously reported cytotoxic and antiprotozoan effects of some cycadales plants motivated us to evaluate the *Z. floridana* A. DC. methanol extract and its different fractions for a potential toxoplasmocidal effect in addition to the cytotoxic potential against breast cancer (MCF-7) and colon cancer (HCT-116) cell lines. Additionally, we aimed to identify the phytochemicals which could be responsible for the resulting activity.

## 2. Materials and Methods

### 2.1. General Experimental Procedures

Solvents used were of HPLC analytical grade ≥ 99.9% and were purchased from Sigma Co. (St. Louis, MO, USA). RPMI-1640 medium reagent, dimethyl sulfoxide (DMSO), 4,5 dimethylthiazole-2-yl-2,5 diphenyltetrazolium bromide (MTT), phosphate buffer saline, trypan blue and doxorubicin HCl were obtained from Sigma Co. (St. Louis, MO, USA). Fetal bovine serum was purchased from Gibco Co. (Carlsbad, CA, USA) and cotrimoxazole (Septrin™ oral suspension) from GlaxoSmithKline.

NMR experiments were performed using a Bruker Avance III spectrometer (Rheinstetten, Germany), with 400 MHz for ^1^H and 100 MHz for APT and DEPT-Q NMR. A UV/Vis spectrophotometer UV-1800 from Shimadzu Co. (Tokyo, Japan) was used to record UV spectra. An FT/IR-6100 spectrophotometer from Jasco Co. (Tokyo, Japan) was used to measure IR spectra as KBr discs. A compact mass spectrometer (CMS) from Advion Co. (New York, NY, USA) was used to record ESI-MS spectra. An ELISA Processor II Microplate Reader EXL800 from Biotek Co. (Winooski, VT, USA) was used for the cytotoxic assay.

Diaion HP-20 from Mitsubishi Chemical Co. (Tokyo, Japan), Sephadex LH-20 was purchased from Sigma-Aldrich Chemical Co. (St. Louis, MO, USA), Silica gel (70–230 mesh) and precoated TLC sheets of silica gel F_254_ were obtained from Merck Co. (Darmstadt, Germany). Authentic samples of bilobetin and amentoflavone for Co-TLC and superimposable IR experiments were provided by the Department of Pharmacognosy, Faculty of Pharmacy, Tanta University, Egypt. FeCl_3_ (5%), AlCl_3_ (5%) and H_2_SO_4_ (10%) spray reagents were used for the detection on TLC. The solvent systems used for TLC were CHCl_3_-MeOH (9:1) “S1”, CHCl_3_-MeOH (8:2) “S1” and CHCl_3_-MeOH-H_2_O (6:4:1) “S3”.

### 2.2. Plant Material 

Leaves of *Z. floridana* A. DC. were collected from El-Abd Garden at 68 kilos from desert Cairo-Alexandria Road in July 2018. It was kindly provided and identified by researcher Rabea Sharawy Agronomist and palm researcher. A voucher sample (No. PGG-013) was deposited at the herbarium of Faculty of Pharmacy, Tanta University, Egypt.

### 2.3. Extraction and Isolation 

The extraction, fractionation and isolation steps are shown in the supplementary material (Figures S1 and S2). The plant material was dried in the shade, reduced to powder, and stored in tightly closed containers. The plant powder (3.4 kg) was extracted with methanol by cold maceration till exhaustion. The total methanol extract was evaporated under reduced pressure at 40 °C to yield a green residue (181.25 g). Methanol extract residue (161.32 g) was suspended in 50% aqueous methanol (750 mL) and successively fractionated with petroleum ether (40–60 °C), chloroform (CHCl_3_), ethyl acetate (EtOAc), and *n*-butanol (*n*-BuOH) to yield 23.72 g, 4.28 g, 5.83 g and 42.10 g, respectively.

The *n*-BuOH fraction (42.10 g) was suspended in a deionized water and applied to Diaion HP-20 column (Φ 5 cm × 28 cm, 200 g). The column was first eluted with (4 L) of deionized water followed by (2 L) 100% MeOH. The methanol fraction was concentrated to give a brown residue (3.3 g) to be used for biological screening and chromatography separation. A silica gel column (Φ 2 cm × 48 cm, 82 g) was used to isolate the components using a gradient elution, starting with 100% CHCl_3_ and the polarity was increased using MeOH. Fractions (10 mL) were collected and similar fractions on TLC were combined to afford five groups of fractions (F1 to F5).

F1 eluted with CHCl_3_:MeOH (95:5) gave a yellow colored residue (172.2 mg) which was chromatographed further on a silica gel column (Φ 1 cm × 17 cm, 6 g) using a gradient elution of CHCl_3_ and MeOH to obtain two subfractions F1-1 to F1-2. F1-1 eluted with CHCl_3_:MeOH (97:3) gave a yellow colored residue (84.1 mg) was re-chromatographed on a Sephadex LH-20 column (Φ 1.5 cm × 25 cm, 20 g) using MeOH (HPLC grade) to give compound (**1**) (9.1 mg).

F2 eluted with CHCl_3_:MeOH (90:10) gave a yellow colored residue (153.4 mg), which was re-chromatographed on a silica gel column (Φ 1 cm × 16.5 cm, 5 g) using CHCl_3_ and MeOH in a gradient elution to give two subfractions F2-1 and F2-2. F2-2 eluted with CHCl_3_:MeOH (93:7) gave a yellow colored residue (80.3 mg) was purified on a Sephadex LH-20 (Φ 1.5 cm × 25 cm, 20 g) using MeOH (HPLC grade) to give compound (**2**) (11.2 mg). 

F3 eluted with CHCl_3_:MeOH (85:15) yielded a yellow colored residue (660.5 mg) which was chromatographed further on a silica gel column (Φ 1.5 cm × 24 cm, 18 g) using a gradient elution of CHCl_3_ and MeOH to afford two subfractions F3-1 to F3-2. F3-1 eluted with CHCl_3_:MeOH (90:10) yielded a yellowish white colored residue (66.3 mg) was purified further using a Sephadex LH-20 (Φ 1.5 cm × 25 cm, 20 g) and MeOH (HPLC grade) to afford compound (**3**) (8.1 mg). F3-2 eluted with CHCl_3_:MeOH (88:12) yielded a yellow colored residue (112.5 mg) was also purified further using a Sephadex LH-20 (Φ 1.5 cm × 25 cm, 20 g) eluted with MeOH (HPLC grade) to afford compound (**4**) (15.1 mg).

F4 eluted with CHCl_3_:MeOH (80:20) produced a light yellow colored residue (500.6 mg). Further chromatography on a silica gel column (Φ 1.5 cm × 20 cm, 15 g) using a gradient elution with CHCl_3_ and MeOH resulted in two subfractions F4-1 to F4-2. F4-2 eluted with CHCl_3_:MeOH (82:18) produced a yellowish white colored residue (92.5 mg) was purified on a Sephadex LH-20 (Φ 1.5 cm × 25 cm, 20 g) using MeOH (HPLC grade) to give compound (**5**) (10.0 mg).

F5 eluted with CHCl_3_:MeOH (60:40) yielded a dark brown colored residue (375.3 mg). Further chromatography on a silica gel column (Φ 1.5 cm × 13 cm, 12 g) using the same gradient elution system afforded two subfractions F5-1 to F5-2. F5-2 eluted with CHCl_3_:MeOH (65:35) yielded a dark yellow colored residue (127.7 mg) was purified on a Sephadex LH-20 (Φ 1.5 cm × 25 cm, 20 g) using MeOH (HPLC grade) to afford compound (**6**) (11.1 mg).

*4′,4′″-O-methyl amentoflavone “Isoginkgetin”* (**1**). Amorphous yellow powder; UV (MeOH) λ_max_: 222, 271 and 328 nm see (Supplementary Material Figure S3); IR (KBr disc) ν_max_ = 3415, 2922, 2853, 1644, 1617, 1431, 1382, 1254, 1172, 1114, 1028, 875, 835, 616, 473, 401, 271, 260 cm^−1^ see (Supplementary Material Figure S4); ^1^H-NMR (CD_3_OD, 400 MHz) δ (ppm) 8.09 (brs, H-6′, 1H), 7.98 (brs, H-2′, 1H), 7.56 (brs, H-2′″, 6′″, 2H), 7.33 (brs, H-5′, 1H), 6.92 (brs, H-3′″, 5′″, 2H), 6.82 (s, H-3, 1H), 6.68 (s, H-3″, 1H), 6.45 (brs, H-8, 1H), 6.40 (s, H-6″, 1H), 6.22 (brs, H-6, 1H), 3.84 (s, OMe-4′, 3H), 3.80 (s, OMe-4′″, 3H), see (Supplementary Material Figure S5A); DEPTQ-NMR (CD_3_OD, 100 MHz) δ (ppm) 182.3 (C-4), 182.0 (C-4″), 164.8 (C-2″), 164.3 (C-2), 164.0 (C-7), 162.8 (C-4′″), 162.1 (C-5), 161.8 (C-7″), 161.1 (C-4′), 161.0 (C-5″), 158.0 (C-9), 154.8 (C-9″), 130.8 (C-6′), 128.0 (C-2′), 127.5 (C-2′″, 6′″), 122.9 (C-1′), 122.0 (C-1′″), 121.6 (C-3′), 114.1 (C-3′″, 5′″), 111.1 (C-5′), 103.9 (C-10, 10″), 103.1 (C-3″), 102.4 (C-3), 98.8 (C-6″), 98.5 (C-6), 93.7 (C-8), 55.0 (4′-OCH_3_), 54.5 (4′″-OCH_3_) see (Supplementary Material Figure S5B); ESIMS: *m*/*z* 589.4 for [M + Na]^+^ and 565.5 for [M − H]^−^ see (Supplementary Material Figure S6).

*Amentoflavone 4′-O-methyl ether “Bilobetin”* (**2**). Amorphous yellow powder; UV (MeOH) λ_max_: 234, 270 and 330 nm see (Supplementary Material Figure S7); IR (KBr disc) ν_max_= 3417, 2922, 2853, 1643, 1616, 1579, 1500, 1432, 1377, 1281, 1248, 1172, 1112, 610, 588, 474, 271, 262, 249, 239 cm^−1^ see (Supplementary Material Figure S8A); ^1^H-NMR (CD_3_OD, 400 MHz) δ (ppm) 8.06 (brd, *J* = 8 Hz, H-6′, 1H), 7.97 (brs, H-2′, 1H), 7.46 (d, *J* = 8 Hz, H-2′″, 6′″, 2H), 7.29 (d, *J* = 8 Hz, H-5′, 1H), 6.73 (d, *J* = 8 Hz, H-3′″, 5′″, 2H), 6.66 (s, H-3″, 1H), 6.61 (s, H-3, 1H), 6.44 (brs, H-8, 1H), 6.37 (s, H-6″, 1H), 6.20 (brs, H-6, 1H), 3.82 (s, OMe-4′, 3H) see (Supplementary Material Figure S9A); APT-NMR (CD_3_OD, 100 MHz) δ (ppm) 182.7 (C-4″), 182.4 (C-4), 164.8 (C-2″), 164.4 (C-7), 164.3 (C-2), 161.9 (C-7″), 161.7 (C-5), 161.2 (C-4′″), 161.0 (C-4′, C-5″), 158.0 (C-9), 154.8 (C-9″), 130.8 (C-6′), 127.9 (C-2′), 127.7 (C-2′″, 6′″), 122.9 (C-1′), 122.0 (C-3′), 121.6 (C-1′″), 115.5 (C-3′″, 5′″), 111.1 (C-5′), 103.8 (C-8″, 10), 103.7 (C-10″), 103.1 (C-3), 101.9 (C-3″), 98.8 (C-6″), 98.4 (C-6), 93.8 (C-8), 55.0 (4′-OCH_3_) see (Supplementary Material Figure S9B); ESIMS: *m*/*z* 575.4 for [M + Na]^+^ and 551.1 for [M − H]^−^ see (Supplementary Material Figure S10).

*Syringic acid* (**3**). Amorphous white powder; UV (MeOH) λ_max_: 234 and 260 nm see (Supplementary Material Figure S11); ^1^H-NMR (CD_3_OD, 400 MHz) δ (ppm) 7.54 (s, H-2, 6, 2H), 3.72 (s, 3,5-OCH_3_, 6H) see (Supplementary Material Figure S12A); APT-NMR (CD_3_OD, 100 MHz) δ (ppm) 168.5 (C-7), 147.3 (C-3, 5), 140.0 (C-4), 120.0 (C-1), 108.8 (C-2, 6), 55.6 (3,5-OCH_3_) see (Supplementary Material Figure S12B); ESIMS: *m*/*z* 199.1 for [M + H]^+^ and 197.1 for [M − H]^−^ see (Supplementary Material Figure S13).

*Amentoflavone* (**4**). Amorphous yellow powder; UV (MeOH) λ_max_: 232, 274 and 329 nm see (Supplementary Material Figure S14); IR (KBr disc) ν_max_ = 3417, 2922, 2853, 1651, 1612, 1574, 1493, 1426, 1360, 1285, 1243, 1167, 1106, 1050, 1028, 834, 637, 588, 561, 258 cm^−1^ see (Supplementary Material Figure S15A); ^1^H-NMR (CD_3_OD, 400 MHz) δ (ppm) 7.85 (brs, H-2′, 1H), 7.73 (brd, *J* = 8 Hz, H-6′, 1H), 7.39 (d, *J* = 8 Hz, H-2′″, 6′″, 2H), 6.97 (d, *J* = 8 Hz, H-5′, 1H), 6.59 (d, *J* = 8 Hz, H-3′″, 5′″, 2H), 6.46 (s, H-3, 1H), 6.45 (s, H-3″, 1H), 6.29 (brs, H-8, 1H), 6.24 (s, H-6″, 1H), 6.06 (brs, H-6, 1H) see (Supplementary Material Figure S16A); APT-NMR (CD_3_OD, 100 MHz) δ (ppm) 182.7 (C-4″), 182.3 (C-4), 164.7 (C-2″), 164.5 (C-2, 7), 162.6 (C-7″), 161.7 (C-5), 161.1 (C-4′″), 161.0 (C-5″), 159.7 (C-4′), 157.9 (C-9), 155.0 (C-9″), 131.3 (C-6′), 127.8 (C-2′″, 6′″), 127.4 (C-2′), 121.7 (C-1′″), 121.6 (C-1′), 120.3 (C-3′), 116.1 (C-5′), 115.4 (C-3′″, 5′″), 104.0 (C-8″), 103.8 (C-10, C-10″), 102.5 (C-3), 101.9 (C-3″), 98.7 (C-6, 6″), 93.7 (C-8) see (Supplementary Material Figure S16B); ESIMS: *m*/*z* 561.4 for [M + Na]^+^ and 537.1 for [M − H]^−^ see (Supplementary Material Figure S17).

*Gallic acid* (**5**). Amorphous white powder; UV (MeOH) λ_max_: 227 and 266 nm see (Supplementary Material Figure S18); IR (KBr disc) ν_max_ = 3494, 3414, 3285, 2922, 2851, 2668, 1645, 1615, 1539, 1426, 1386, 1319, 1269, 1217, 1029, 732, 631, 557, 486, 266 cm^−1^ see (Supplementary Material Figure S19); ^1^H-NMR (CD_3_OD, 400 MHz) δ (ppm) 6.93 (s, H-2, 6, 2H) see (Supplementary Material Figure S20A); APT-NMR (CD_3_OD, 100 MHz) δ (ppm) 167.6 (C-7), 145.1 (C-3, 5), 138.4 (C-4), 120.0 (C-1), 108.6 (C-2, 6) see (Supplementary Material Figure S20B); ESIMS: *m*/*z* 171.1 for [M + H]^+^ and 169.1 for [M − H]^−^ see (Supplementary Material Figure S21).

*Apigenin 6, 8-di-C-β-D glucoside “Vicenin-2”* (**6**). Amorphous yellow powder; UV (MeOH) λ_max_: 241, 265 and 322 nm see (Supplementary Material Figure S22); IR (KBr disc) ν_max_ = 3416, 2921, 2852, 1652, 1621, 1430, 1385, 1113, 877, 833, 618, 475, 402, 307, 272, 254 cm^−1^ see (Supplementary Material Figure S23); ^1^H-NMR (CD_3_OD, 400 MHz) δ (ppm) 7.99 (d, *J* = 8 Hz, H-2′, 6′, 2H), 6.95 (d, *J* = 8 Hz, H-3′, 5′, 2H), 6.64 (s, H-3, 1H), 5.05 (d, *J* = 9.6 Hz, Glu H-1′″, 1H), 5.01 (d, *J* = 9.6 Hz, Glu H-1″, 1H), 4.12 (m, Glu H-2″, 1H), 3.97 (m, Glu H-6′″, 2H), 3.88 (m, Glu H-6″, 2H), 3.71 (m, Glu H-2′″, 1H), 3.68 (m, Glu H-4′″, 1H), 3.59 (m, Glu H-3′″, 1H), 3.58 (m, Glu H-3″, 1H), 3.57 (m, Glu H-4″, 1H), 3.48 (m, Glu H-3′″, 1H), 3.44 (m, Glu H-5″, 1H) see (Supplementary Material Figure S24A); APT-NMR (CD_3_OD, 100 MHz) δ (ppm) 182.8 (C-4), 165.3 (C-2), 161.4 (C-7, 4′), 159.1 (C-5), 156.2 (C-9), 128.7 (C-2′, 6′), 122.0 (C-1′), 115.6 (C-3′, 5′), 108.3 (C-6), 104.7 (C-8), 104.6 (C-10), 102.4 (C-3), 81.5 (Glu C-5′″), 81.1 (Glu C-5″), 78.8 (Glu C-3′″), 77.7 (Glu C-3″), 74.8 (Glu C-1″), 73.7 (Glu C-1′″), 72.3 (Glu C-2′″), 71.7 (Glu C-2″), 70.9 (Glu C-4′″), 69.5 (Glu C-4″), 61.6 (Glu C-6′″), 60.3 (Glu C-6″) see (Supplementary Material Figure S24B); HSQC and HMBC NMR (CD_3_OD) see (Supplementary Material Figure S25); ESIMS: *m*/*z* 617.1 for [M + Na]^+^ and 593.1 for [M − H]^−^ see (Supplementary Material Figure S26).

### 2.4. Biological Activity

#### 2.4.1. Toxoplasmocidal Activity

A virulent RH strain of *T. gondii* was supplied by the Medical Parasitology Department of the Faculty of Medicine (Alexandria University, Egypt) for this experiment. According to the method reported by Kavitha et al., 2012, different concentrations of the total methanol extract of *Z. floridana* leaves and its different fractions were tested for toxoplasmocidal activity [16]. The mean effective concentration (EC_50_) was calculated as μg/mL and compared to that of cotrimoxazole as a positive control drug. 

#### 2.4.2. Cytotoxic Activity 

Hepatocellular carcinoma (HEPG-2), mammary gland breast carcinoma (MCF-7), colorectal carcinoma (HCT-116), prostate carcinoma (PC-3), cervical carcinoma (HELA) and the normal (WISH) amniotic cell lines were obtained from the American Type Culture Collection (ATCC) via VACSERA Company (Cairo, Egypt)**.**

Using MTT assay method, the cytotoxicity assay was carried out in accordance with the reported procedures [17,18,19,20]. Seven different concentrations (1.56, 3.125, 6.25, 12.5, 25, 50 and 100 μg/mL) of *Z. floridana* total methanol extract dissolved in DMSO, were tested against the investigated cancer cell lines as well as one normal cell line (WISH) to test the safety of the plant extract on the normal cells. Then, the most affected cell lines were incubated with different concentrations of petroleum ether, CHCl_3_, EtOAc and *n*-BuOH fractions using doxorubicin as reference drug. IC_50_ was calculated and the cytotoxic potency was assessed according to the classification of Hossan and Abu Melha, 2014 [21].

### 2.5. In Silico Molecular Docking Studies

Molecular docking studies (by MOE 2020.9010 version) were carried out to show the binding mode and interactions of the isolated molecules (**1**–**6**) (by Discovery Studio (DS) visualizer program). The Protein Data Bank was used to obtain the crystal structure of TS inhibitor in association with *T. gondii* TS-DHFR, which has a resolution of 2.79 Å (PDB ID: 4KY4) [22] and CDK2 in association with inhibitor, which has a resolution of 2.20 Å (PDB ID: 1FVT) [23]. We chose a single chain (A) pre-docked with its unique ligand “2-amino-5-(phenylsulfanyl)-3,9-dihydro-4H-pyrimido [4,5-b]indol-4-one” (1UE) and “4-[(2Z)-2-(5-bromo-2-oxo-1,2-dihydro-3H-indol-3-ylidene) hydrazinyl]benzene-1-sulfonamide” (**106**) for TS-DHFR and CDK2, respectively. Both hydrophobic and hydrophilic amino acids were found in the ligand-binding site of the relevant enzymes. At the active sites, (1UE and 106) exhibited both hydrophilic and hydrophobic interactions. The redocking method for the ligands (1UE and 106) was performed with the goal of validating the docking protocol by creating numerous docked poses, one docked pose for each ligand had an RMSD value less than 1 (i.e., 0.8118 and 0.9501 Å for 1UE and 106, respectively), thus confirming the docking procedure. The molecular docking investigation demonstrated that all of the compounds tested fit well into the enzymes’ active pockets. Furthermore, based on the results of the binding free energy calculation, the most promising docked conformations of each isolate were analyzed further for binding mode analysis.

### 2.6. Statistical Analysis

All experiments were carried out at least three times, the data are expressed as the mean ± standard error of the mean (SEM).

## 3. Results

### 3.1. Biological Activity

#### 3.1.1. Toxoplasmocidal Activity

**Figure 1:**
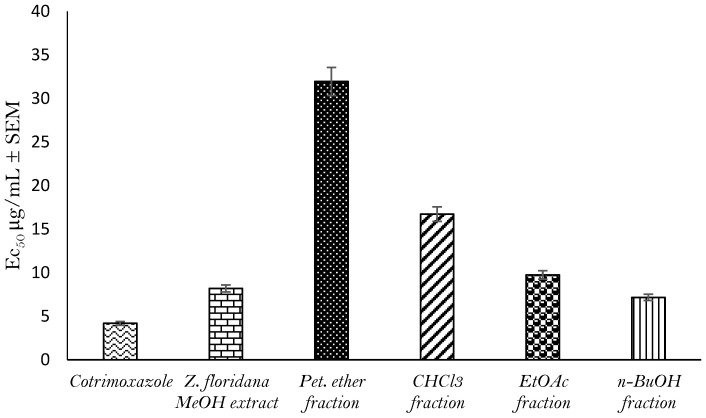
Toxoplasmocidal effect (EC_50_ ± SEM) of *Z. floridana* methanol extract and its different fractions against *T. gondii*.

*Z. floridana* leaves’ total methanol extract and its different fractions were screened for toxoplasmocidal activity against *T. gondii* RH strain tachyzoites. The relative mortality of the parasite incubated with different concentrations of the tested extracts was assessed using trypan blue dye. The results revealed that *Z. foridana* showed a potent toxoplasmocidal activity with an EC_50_ of 8.19 μg/mL compared to that of cotrimoxazole standard drug (EC_50_ of 4.18 ± 0.3 μg/mL). Moreover, the *n*-BuOH fraction showed the highest toxoplasmocidal activity followed by the EtOAc fraction then the CHCl_3_ fraction and finally the pet-ether fraction with EC_50_ of 7.16 ± 0.4, 9.74 ± 0.5, 16.71 ± 0.8 and 31.95 ± 1.3 μg/mL, respectively. (Figure 1, Supplementary Material Table S1)

#### 3.1.2. Cytotoxic Activity

**Figure 2:**
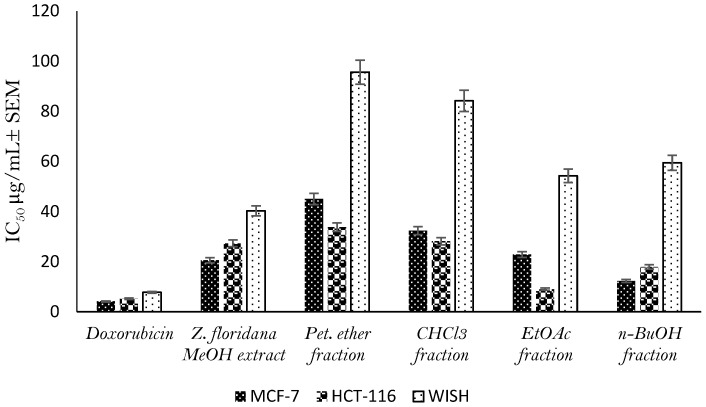
Cytotoxic effect (IC_50_ ± SEM) of *Z. floridana* methanol extract and its different fractions against different cell lines.

The cytotoxic activity of *Z. floridana* leaves methanol extract and its different fractions was evaluated using the MTT assay protocol. The percent inhibition of the cancer cells’ viability under the effect of the different tested concentrations is shown in the Supplementary Material Tables S2 and S3. The results showed that total methanol extract of *Z. floridana* has a cytotoxic potential against MCF-7 and HCT-116 cell lines with IC_50_ of 20.57 ± 1.7 and 27.33 ± 2.3 μg/mL, respectively, compared to that of doxorubicin as a positive control drug (IC_50_ of 4.17 ± 0.2 and 5.23 ± 0.3 μg/mL). Interestingly, *Z. floridana* methanol extract showed a low cytotoxicity effect against normal cell line (WISH) with an IC_50_ of 40.29 ± 3.2 μg/mL (Supplementary Material Table S4). Amongst tested fractions, the EtOAc and the *n*-BuOH fractions showed highest cytotoxic potential against MCF-7 and HCT-116 cell lines with IC_50_ of 22.89 ± 1.8 and 9.04 ± 0.8 μg/mL, respectively, for the EtOAc fraction and IC_50_ of 12.33 ± 1.1 and 17.88 ± 1.4 μg/mL, respectively, for the *n*-BuOH fraction. (Figure 2, Supplementary Material Table S5).

### 3.2. Phytochemical Investigation

The *n*-BuOH fraction was subjected to several chromatographic columns to separate six compounds (**1**–**6**) (Figure 3). These compounds are 4′,4′″-*O*-methyl amentoflavone (**1**), amentoflavone 4′-*O*-methyl ether (**2**), syringic acid (**3**), amentoflavone (**4**), gallic acid (**5**) and apigenin 6, 8-di-*C*-*β*-D glucopyranoside (**6**). Compounds (**1**, **3**, **5** and **6**) were isolated for the first time from *Z. floridana*. The structures of compounds (**1**–**6**) were elucidated by a variety of spectroscopies including (UV, IR, ESIMS, ^1^H, APT, DEPTQ, HSQC, and HMBC NMR) and compared to the available authentic compounds and the published data. The spectra of the IR, UV, mass, and NMR analysis of all the isolated compounds are provided in the Supplementary Material Figures S3–S26.

#### Identification of the Compounds (**1**–**6**)

Compound (**1**) was isolated as an amorphous yellow powder. It gave a yellow color with 5% AlCl_3_ and a UV λ_max_ at 222, 271 and 328 nm, which suggested that compound (**1**) is a flavonoid. The IR spectrum showed a strong band at 3415 cm^−1^ for phenolic hydroxyl (OH) groups stretching and at 1644 cm^−1^ for a carbonyl (C=O) group. The ^1^H-NMR spectrum of compound (**1**) proposed a biflavonoid structure consisting of two units (I and II). The ^1^H-NMR spectrum of compound (**1**) showed an AA’BB’ coupling system of the *para* substituted ring B of unit II at δ_H_ 6.92 (2H, brs, H-3′″, H-5′″) and 7.56 (2H, brs, H-2′″, H-6′″). In addition, the ^1^H-NMR spectrum showed an ABX coupling system at δ_H_ 7.33 (1H, brs, H-5′), 7.98 (1H, brs, H-2′) and 8.09 (1H, brs, H-6′) of ring B of unit I indicating that C-3′ was the position of linkage of the two flavonoid units. Signals for the two *meta*-coupled protons at δ_H_ 6.22 (1H, brs, H-6) and 6.45 (1H, brs, H-8) were ascribed to ring A of unit I. The DEPT-Q NMR of compound (**1**) showed a downfield shift for C-3′, C-8″ signals at δ_DEPT-Q_ 121.6 and 104.2, respectively compared to the apigenin ^13^C-NMR spectral data [24]. The ^1^H-NMR signal at δ_H_ 6.40 (1H, s, H-6″) indicated that there is no *meta* coupling between H-6″, H-8″, all of these signals support the interflavonoid linkage between C-3′ and C-8″. Therefore, compound (**1**) structure was suggested as a 3′, 8″ biapigenin structure. The ^1^H, DEPT-Q NMR spectrum also showed signals at δ_H_ 3.80, 3.84 and at δ_DEPT-Q_ 54.5 and 55.0 that are belonging to (3H, s, 4′″-OMe) and (3H, s, 4′-OMe), respectively. The location of the methoxy group was proposed at C-4′ and C-4′″ due to the upfield shift of ∆δ 4.6 ppm at C-5′ (δ_DEPT-Q_ 111.1) and downfield shift of ∆δ 2 ppm at C-1′ (δ_DEPT-Q_ 122.9) in addition to the upfield shift of ∆δ 1.6 ppm at C-3′″, 5′″ (δ_DEPT-Q_ 114.1) and downfield shift of ∆δ 1.2 ppm at C-1′″ (δ_DEPT-Q_ 122.0) compared to the apigenin ^13^C-NMR spectral data [24]. The ESIMS of (**1**) showed a pseudo molecular ion at *m*/*z* 589.4 for [M + Na]^+^ with a molecular formula of C_32_H_22_O_10_Na, and at *m*/*z* 565.5 for [M − H]^−^ with a molecular formula of C_32_H_21_O_10_ suggesting a molecular formula for (**1**) as C_32_H_22_O_10_, which is consistent with an amentoflavone di-methoxy derivative. By comparing all the spectral data of compound (**1**) to those reported in the literature [4,24,25,26], compound (**1**) was identified as 4′,4′″-*O*-methyl amentoflavone (isoginkgetin). This is the first report of isoginkgetin from *Z. floridana*.

Compound (**2**) was isolated as an amorphous yellow powder. It gave a yellow color with 5% AlCl_3_ and a UV λ_max_ at 234, 270 and 330 nm, which suggested that compound (**2**), is a flavonoid compound. The IR spectrum showed a strong band at 3417 cm^−1^ for phenolic hydroxyl (OH) groups stretching and at 1643 cm^−1^ for a carbonyl (C=O) group. The ^1^H NMR spectrum of compound (**2**) showed the pattern of a biflovonoid pattern as in compound (**1**). The ^1^H NMR spectrum of compound (**2**) exhibited An AA’BB’ coupling system of the *para*-substituted ring B of unit II at δ_H_ 6.73 (2H, d, *J* = 8 Hz, H-3′″, H-5′″) and 7.46 (2H, d, *J* = 8 Hz, H-2′″, H-6′″). Additionally, an ABX coupling system at δ_H_ 7.29 (1H, d, *J* = 8 Hz, H-5′), 7.97 (1H, brs, H-2′) and 8.06 (1H, brd, *J* = 8 Hz, H-6′) of ring B of unit I was observed indicating that C-3′ was the position of the linkage of the two flavonoid units. Signals for two *meta*-coupled protons at δ_H_ 6.20 (1H, brs, H-6) and 6.44 (1H, brs, H-8) of ring A of unit I were present. The APT NMR of compound (**2**) showed a downfield shift for C-3′, C-8″ signals at δ_APT_ 122.0 and 103.8, respectively, compared to the apigenin ^13^C-NMR spectral data [24]. The ^1^H NMR signal at δ_H_ 6.37 (1H, s, H-6″) indicated that there is no *meta* coupling between H-6″, H-8″, these signals support the interflavonoid linkage between C-3′ and C-8″. Therefore, a 3′, 8″ biapigenin structure was proposed for compound (**2**). Signals at δ_H_ 3.82 (3H) and at δ_APT_ 55.0 were also observed indicating a methoxy group. The location of the methoxy group was confirmed at C-4′ due to the upfield shifts of ∆δ 4.6 ppm at C-5′ (δ_APT_ 111.1) and the downfield shift of ∆δ 2 ppm at C-1′ (δ_APT_ 122.9) compared to the apigenin ^13^C NMR spectral data [24]. The ESIMS of (**2**) showed a pseudo molecular ion at *m*/*z* 575.4 for [M + Na]^+^ and 551.1 for [M − H]^−^ suggesting a molecular formula for (**2**) as C_31_H_20_O_10_ which is consistent with an amentoflavone methoxy derivative. The IR spectrum of compound (**2**) was found identical to an authentic sample of bilobetin (Figure S8B). By comparing our data to those reported in the literature [2,24,27,28], compound (**2**) was identified as amentoflavone 4′-*O*-methyl ether (bilobetin).

Compound (**3**) was isolated as an amorphous white powder. It gave a blue color with FeCl_3_ spray reagent and a UV λ_max_ at 234 and 260 nm suggesting that compound (**3**) has a phenolic acid nucleus. The ^1^H NMR spectrum of compound (**3**) showed a typical signal for two symmetric aromatic protons at δ_H_ 7.54 (2H, s, H-2, H-6) which suggested that this compound has 1,3,4,5-tetra-substituted aromatic ring. Another signal at δ_H_ 3.72 integrating for 6 carbons (6H, s, 3, 5-OCH_3_) indicated the presence of two methoxy groups in this compound. The APT-NMR spectrum showed the presence of two equivalent olefinic methine carbons and two equivalent methoxy carbons at δ_APT_ (108.8, 55.6), respectively. Additionally, the APT-NMR spectrum showed five quaternary carbon signals including three oxygenated olefinic carbons two of them are equivalent at δ_APT_ (147.3, 140.0) and were assigned to C-3, 5 and C-4, respectively. Another signal at δ_APT_ (168.5) indicated the presence of carboxyl carbon (C-7) and a signal at δ_APT_ (120.0) for the aromatic carbon C-1. The ESIMS of compound (**3**) showed a pseudo molecular ion at *m*/*z* 199.1 for [M + H]^+^ with a molecular formula C_9_H_11_O_5_, 197.1 for [M − H]^−^, which is consistent with syringic acid. All of these spectral data were identical to those previously reported of syringic acid [29,30,31]. This is the first report of syringic acid from *Z. floridana*.

Compound (**4**) was obtained as an amorphous yellow powder. It gave a yellow color with 5% AlCl_3_ and UV λ_max_ at 232, 274 and 329 nm suggesting that compound (**4**) is a flavonoid structure. The IR spectrum showed a strong band at 3417 cm^−1^ for phenolic hydroxyl (OH) groups stretching and at 1651 cm^−1^ for a carbonyl (C=O) group. The APT NMR analysis showed signals for 30 carbons, including two carbonyl group signals at δ_APT_ 182.3 and 182.7 of (C-4, C-4′, respectively). These signals suggest that compound (**4**) is a biflavonoid. The ^1^H NMR data showed typical signals for AA’BB’ coupling pattern at δ_H_ 7.39 (2H, d, *J* = 8 Hz, H-2′″, 6′″) and 6.59 (2H, d, *J* = 8 Hz, H-3′″, 5′″), which suggested the presence of 1, 4-disubstituted benzene ring B of unit II and typical signals for an ABX coupling system at δ_H_ 6.97 (1H, d, *J* = 8 Hz, H-5′), 7.73 (1H, brd, *J* = 8 Hz, H-6′) and 7.85 (1H, brs, H-2′) of ring B of unit II suggesting that C-3′ was the position of linkage of the two flavonoid units. Signals at δ_H_ 6.06 (1H, brs, H-6) and 6.29 (1H, brs, H-8) indicated a *meta* coupling of H-6, H-8 of ring A of unit I. Additionally, only one aromatic proton singlet at δ_H_ 6.24 was assigned to H-6″ with the absence of the proton signal for C-8″ suggested that C-8″ is involved in the interflavonoid linkage. These proton signals in addition to the downfield shift of C-3′ and C-8″ which appeared at δ_APT_ 120.3 and 104.0, respectively, compared to the apigenin ^13^C-NMR spectral data [24], suggested that C-3′ and C-8″ were involved in the linkage between the two flavonoids moieties of the biflavonoid structure which is consistent with amentoflavone in the literature [24,32]. The ESIMS of (**4**) showed a pseudo molecular ion at *m*/*z* 561.4 for [M + Na]^+^ with a molecular formula C_30_H_18_O_10_Na, and at *m*/*z* 537.1 for [M − H]^−^ with a molecular formula C_30_H_17_O_10_ suggesting a molecular formula for compound (**4**) as C_30_H_18_O_10_, which matches amentoflavone. The IR spectrum of compound (**4**) was found identical to an authentic sample of amentoflavone (Figure S15B). The spectral data of compound (**4**) was identical to those reported in the literature for amentoflavone [2,24,32].

Compound (**5**) was isolated as an amorphous white powder. It gave a blue color with FeCl_3_ spray reagent and a UV λ_max_ at 227 and 266 nm suggesting that compound (**5**) has a phenolic acid nucleus. The IR spectrum indicated the presence of a carboxylic group through a strong band at 3494 cm^−1^, hydroxyl phenolic groups at 3414 and 3285 cm^−1^, a carbonyl group at 1645 cm^−1^. The ^1^H NMR spectrum of compound (**5**) showed a singlet integrating for two protons of two similar methine carbons in the aromatic range at δ_H_ 6.93 which suggested that this compound has 1,3,4,5-tetra-substituted aromatic ring similar to compound (**3**). The APT-NMR spectrum showed five signals for seven carbons including a carbonyl carbon at δ_APT_ 167.6 (C=O), 3 oxygenated quaternary carbons at δ_APT_ 145.1 (C-3, 5) and 138.4 (C-4), a signal for two equivalent methine carbons at δ_APT_ 108.6 (C-2, 6) and another quaternary carbon signal at δ_APT_ 120.0 (C-1). This pattern proposed 3, 4, 5-trihydroxy benzoic acid which is known as gallic acid. The ESIMS of compound (**5**) showed a pseudo molecular ion at *m*/*z* 171.1 for [M + H]^+^ with a molecular formula C_7_H_7_O_5_, and at *m*/*z* 169.1 for [M − H]^−^ with a molecular formula C_7_H_5_O_5_ suggesting a molecular formula for compound (**5**) as C_7_H_6_O_5_, which is consistent with gallic acid. All of these spectral data were identical to the previous literature of gallic acid [33,34,35]. This is the first report of gallic acid from *Z. floridana*.

Compound (**6**) was isolated as an amorphous yellow powder. It gave a yellowish green color with 5% AlCl_3_ spray reagent and brown color with 10% H_2_SO_4_ spray reagent and a UV λ_max_ at 241, 265 and 322 nm suggesting that compound (**6**) is a flavonoid glycoside. The IR spectrum showed a strong band at 3416 cm^−1^ for phenolic hydroxyl (OH) groups stretching and at 1652 cm^−1^ for a carbonyl (C=O) group. The ^1^H NMR data showed typical signals for an AA’BB’ coupling pattern at δ_H_ 7.99 (2H, d, *J* = 8 Hz, H-2′, 6′) and 6.95 (2H, d, *J* = 8 Hz, H-3′, 5′), which suggested the presence of 1, 4-disubstituted benzene ring B. In addition, a singlet at δ_H_ 6.64 (1H, s, H-3) was observed in the ^1^H-NMR spectrum, which indicated that compound (**6**) is a flavone. The appearance of two anomeric doublets signals at δ_H_ 5.01 (d, *J* = 9.6 Hz, Glu H-1″, 1H) and 5.05 (d, *J* = 9.6 Hz, Glu H-1′″, 1H) also, the presence of other sugar signals in the range of δ_H_ 3.44–4.12 suggested the presence of two sugar moieties. In addition, the absence of the proton signals for H-6 and H-8 suggested that the two sugar moieties are linked to C-6 and C-8. The APT-NMR spectrum showed signals for 27 carbons including 12 signals for the two sugars moieties. The two anomeric carbons were assigned at δ_APT_ 74.8 and 73.7 through their correlation to signals at δ_H_ 5.01 and 5.05 in the HSQC spectrum, which suggested the C-glycosidic linkage of these two sugars molecules. The other sugar resonances were identical to glucose moiety [36]. The aglycon carbon signals were assigned with using the HSQC and HMBC experiments which are identical to that of an apigenin moiety [24]. However, the downfield shift of C-6 and C-8 of compound (**6**) compared to that of apigenin supported that these two carbons are linked to sugars moieties. The HMBC correlation data confirmed the linkage of the two glucose moieties at C-6 and C-8 through the correlation of an anomeric proton signal at δ_H_ 5.01 and δ_C_ 108.3 (C-6), 159.1 (C-5), 104.6 (C-10), while the other anomeric proton signal at δ_H_ 5.05 was correlated to δ_C_ 104.7 (C-8) and 156.2 (C-9). The ESI-MS of (**6**) showed pseudo molecular ions at *m*/*z* 617.1 for [M + Na]^+^ with molecular formula C_27_H_30_O_15_Na, and at *m*/*z* 593.1 for [M − H]^−^ with a molecular formula C_27_H_29_O_15_ suggesting a molecular formula for compound (**6**) as C_27_H_30_O_15_. The configuration of glucose at the glycosidic bonds was determined as *β* based on the large *J* value for the anomeric protons (9.6 Hz) and by comparing the resonances of the carbons and protons, the HSQC and the HMBC correlations to the published data of vicenin-2 [37,38]. Based on these data, compound (**6**) was identified as apigenin 6, 8-di-*C*-*β*-D glucoside (vicenin-2). This is the first report of vicenin-2 from *Z. floridana*.

### 3.3. Investigation of the Toxoplasmocidal Effect of Compounds (***1***–***6***) via In Silico Studies

In silico molecular docking studies were carried out for these pure compounds to study their possible toxoplasmocidal and cytotoxic mechanisms. *T. gondii* has a number of viable targets that can be inhibited by several drugs [39,40]. Thymidylate synthase-dihydrofolate reductase (TS-DHFR) was selected as a target for drugs that can eradicate this parasite [41,42,43] and 1UE ligand (the co-crystalized ligand inside active site) was chosen as a positive control compound. According to what was previously published, we found that there is a direct relationship between the inhibition of cyclin dependent kinase 2 (CDK2) and flavonoids in cancer therapy [44,45,46,47]. Thus, we selected (CDK2) as a target protein for a molecular docking cytotoxic evaluation in comparison to the ligand (**106**) (the co-crystalized ligand inside active site) as a positive control compound. Docking results, binding modes, and the interactions of pure compounds isolated from *Z. floridana n*-BuOH fraction with the critical amino acids in the active site of TS-DHFR (PDB ID: 4KY4) and CDK2 (PDB ID: 1FVT) are recorded in Table 1 and Table 2, respectively, and in the Supplementary Material Figures S27–S40.

## 4. Discussion

**Figure 4:**
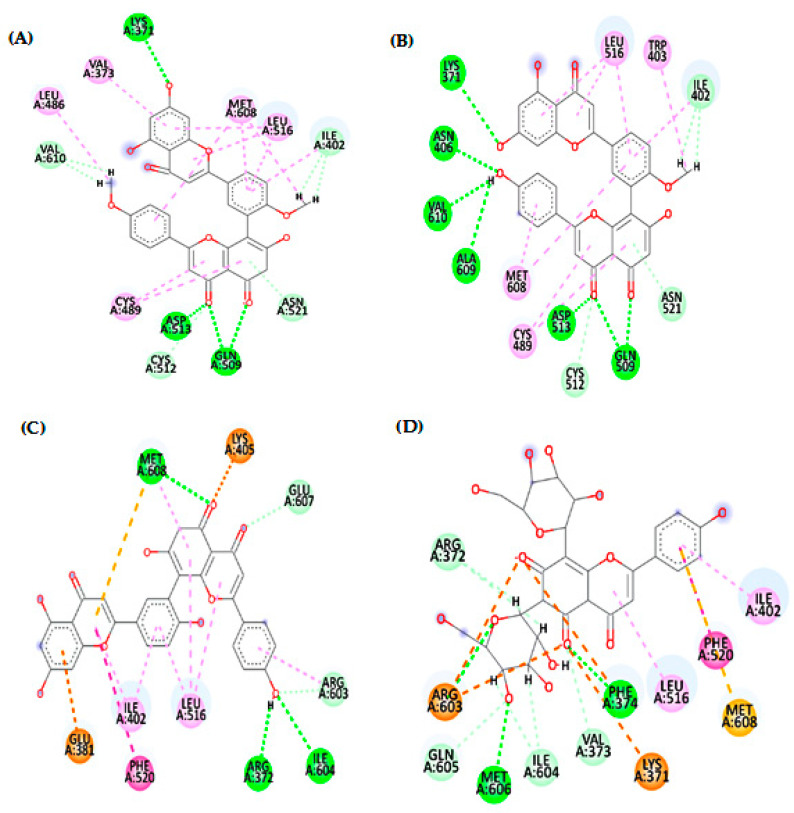
2D patterns demonstrating the binding interaction of compounds **1** (**A**), **2** (**B**), **4** (**C**) and **6** (**D**) into the active site of TS-DHFR (PDB code: 4KY4); dotted pink, violet and orange lines indicate the hydrophobic interaction, and dotted green lines indicate the hydrogen bonds.

**Figure 5:**
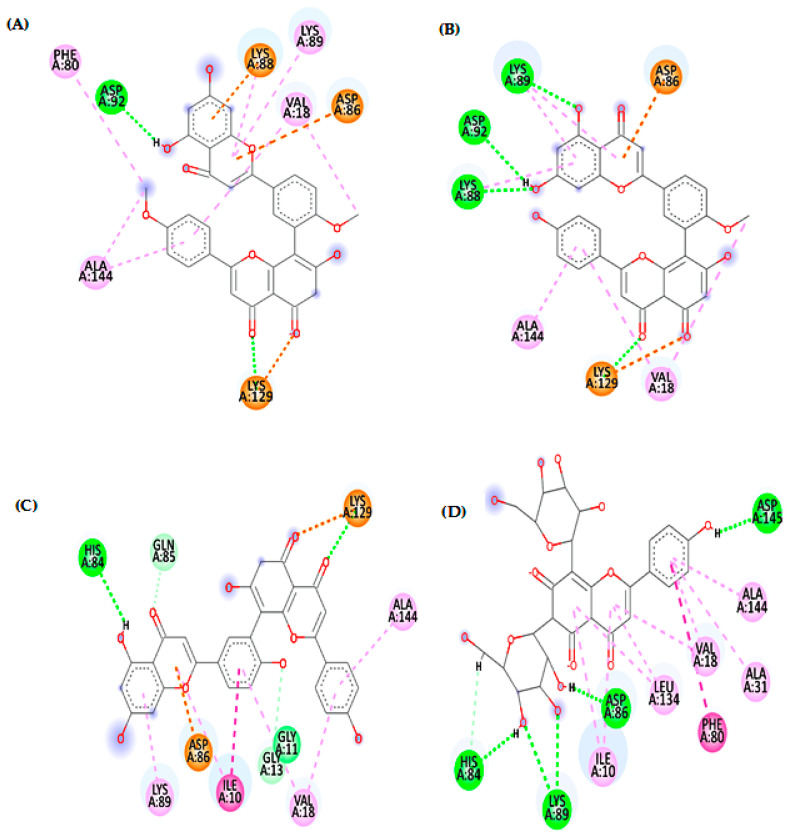
2D patterns demonstrating the binding interaction of compounds **1** (**A**), **2** (**B**), **4** (**C**) and **6** (**D**) into the active site of CDK-2 (PDB ID: 1FVT); dotted pink, violet and orange lines indicate the hydrophobic interaction, and dotted green lines indicate the hydrogen bonds.

This study reports an in vitro assessment for the potential toxoplasmocidal and cytotoxic activities of *Z. floridana* leaves for the first time. The results revealed that the methanol extract of *Z. floridana* leaves showed a significant toxoplasmocidal effect against *T. gondii* tachyzoites, however is less potent compared to a cotrimoxazole drug. Therefore, its different fractions were also tested, and the results showed that *n*-BuOH fraction was the most potent fraction against *T. gondii* but less potent than cotrimoxazole. In addition, *Z. floridana* methanol extract showed moderate cytotoxic activity against MCF-7 and HCT-116 according to the classification of Hossan and Abu Melha, 2014 [21], compared to a doxorubicin drug as a positive control. Interestingly, the total methanol extract showed more selectivity to cancer cells rather than normal cells than doxorubicin. The different fractions of *Z. floridana* extract were tested against the most affected cell lines (MCF-7 and HCT-116). The results showed that the EtOAc and *n*-BuOH fractions were the most potent fractions against the tested cell lines. The EtOAc fraction of *Z. floridana* has very strong cytotoxic activity against HCT-116 and moderate cytotoxic activity against MCF-7, while *n*-BuOH fraction showed strong cytotoxic activity against the two tested cell lines. Based on this biological evaluation, *n*-BuOH fraction is the most potent fraction amongst the tested plant extracts but less potent than doxorubicin. Thus, this motivated us to investigate this fraction for its phytochemicals that may be responsible for these biological activities. The phytochemical investigation of *n*-BuOH fraction of *Z. floridana* led to the isolation of six compounds identified as isoginkgetin, bilobetin, syringic acid, amentoflavone, gallic acid, and vicenin-2. Four compounds of them were isolated for the first time from *Z. floridana* leaves. These compounds were tested previously in other studies against breast and colon cell lines in vitro and they showed cytotoxic effects [2,48,49,50,51,52,53,54,55,56,57], which could account for the cytotoxic effect of the *n*-butanol fraction of *Z. floridana.* The potent toxoplasmocidal effect of *n*-BuOH fraction encouraged us to investigate and predict the possible mechanisms of these compounds to inhibit *T. gondii* using in silico molecular docking study. The results indicated that compound (**2**) “bilobetin” followed by compound (**6**) “vicenin-2”, then compound (**1**) “isoginkgetin”, and finally compound (**4**) “amentoflavone”, had the highest binding affinity to the target protein compared to (1UE). According to the docking scores of bilobetin, isoginkgetin and amentoflavone (−8.95, −8.54 and −7.63 kcal/mol), respectively, the presence of one methoxy group in bilobetin at the central phenyl ring at position 4′ and two methoxy groups in isoginkgetin increase binding affinity over the presence of a hydroxyl group amentoflavone at that position. Where the bilobetin methoxy group makes hydrophobic interaction (with Trp403) and isoginkgetin methoxy groups make hydrophobic interaction (with Leu486 and Met608), but the amentoflavone hydroxyl group did not undergo. Additionally, one of the reasons for the high value of the docking score for bilobetin is that it makes twice the number of hydrogen bonds (6 H-bonds with Lys371, Asn406, Gln509, Asp513, Ala609, and Val610) that isoginkgetin and amentoflavone do (3 H-bonds for each), as shown in Figure 4. Additionally, “vicenin-2” made three hydrogen bonds through hydroxyl groups and oxygen atom in the two glucopyranose rings, which proves the importance of the glucopyranose rings [58]. Further, the phenolic and flavone rings of vicenin-2 had seven hydrophobic interactions with Lys371, Phe374, Ile402, Leu516, Phe520, Arg603, and Met608, as shown in Figure 4. Additionally, the previously published data of cytotoxic activities of the isolated six compounds from *n*-BuOH fraction of *Z. floridana* leaves motivated us to study and predict the possible anticancer mechanism of these compounds. Thus, we performed a molecular docking study for these isolates. The docking results revealed that compound (**6**) “vicenin-2” followed by compound (**1**) “isoginkgetin”, then compound (**4**) “amentoflavone”, and finally compound (**2**) “bilobetin”, had the highest binding affinity to the target protein compared to the ligand (**106**). The ability of vicenin-2 (docking score, −8.38 kcal/mol) to form six hydrogen bonds with four residues (His84, Asp86, Lys89, and Asp145), three of them interacting with the hydroxyl groups in one glucopyranose ring and one hydrogen bond with the phenolic ring, may be the reason for its excellent inhibitory activity [58]. Additionally, Ile10, Val18, Ala31, Phe80, Leu134, and Ala144 exhibited hydrophobic interactions with the phenolic and flavone rings (Figure 5). The almost similar docking scores of isoginkgetin, amentoflavone, and bilobetin (−7.62, −7.60 and −7.58 kcal/mol, respectively), is due to their ability to make from two to four hydrogen bonds and hydrophobic interactions with at least four amino acids (Figure 5). Moreover, these computed binding energy values also confirm and support the in vitro results of the *n*-BuOH effectivity against *T. gondii* and the different cancer cell lines tested.

## 5. Conclusions

In conclusion, the biological screening for *Z. floridana* methanol extracts and its different fractions indicated that although it is less potent than the control drugs, *n*-BuOH fraction has noticeable toxoplasmocidal and cytotoxic activities against two different cell lines. Therefore, the phytochemical investigation of the *n*-BuOH fraction of *Z. floridana* leaves was carried out and resulted in the isolation of six compounds, four of them were isolated for the first time from *Z. floridana* leaves. Various spectroscopies were used to identify these chemicals, and the results were compared to published data. An in silico molecular docking study was used to study the possible toxoplasmocidal and cytotoxic mechanisms of these isolated compounds. The results showed that among all compounds, compounds (**1**, **2**, **4,** and **6**) have the highest docking score. Future research is required to assess these actions in vivo.

## Figures and Tables

**Figure 3:**
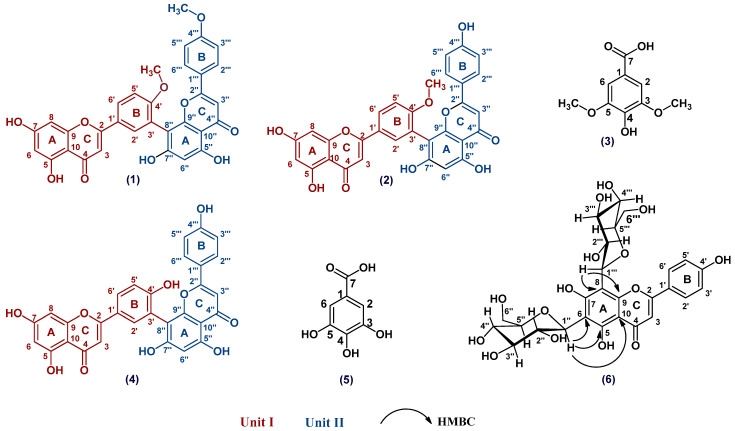
Structures of the compounds (**1**–**6**) isolated from the *n*-BuOH fraction of *Z. floridana* A. DC.

**Table 1:** Docking results, binding modes and interactions of pure compounds isolated from *Z. floridana n*-BuOH fraction in the active site of TS-DHFR (PDB ID: 4KY4).

Compound	Docking Score (kcal/mol)	H-bond Interaction		Hydrophobic Interaction	
		Amino Acid	Fragment	Amino Acid	Fragment
1UE	−6.51	Asn406	Indole ring	Ile402	Indole ring Thiophenol ring
		Asp513	Pyrimidine ring NH_2_	Trp403	Indole ring
		Ala609	NH_2_	Leu516	Thiophenol ring
				Phe520	Thiophenol ring
				Met608	Indole ring Pyrimidine ring
Compound (**1**)	−8.54	Lys371	Flavone ring	Val373	Flavone ring
		Gln509	Flavone ring	Ile402	Phenoxy ring
		Asp513	Flavone ring	Leu486	Methoxy group
				Cys489	Flavone ring
				Leu516	Flavone ring Phenoxy ring
				Met608	Phenoxy ring Methoxy group
Compound (**2**)	−8.95	Lys371	Flavone ring	Ile402	Phenoxy ring
		Asn406	Phenolic ring	Trp403	Phenoxy ring
		Gln509	Flavone ring	Cys489	Flavone ring
		Asp513	Flavone ring	Leu516	Flavone ring Phenoxy ring
		Ala609	Phenolic ring	Met608	Phenolic ring Phenoxy ring
		Val610	Phenolic ring		
Compound (**3**)	−5.15	Gln509	Carboxylic group	Cys489	Methoxy group Phenyl ring
		Asp513	Carboxylic group	Met608	Methoxy group
		Asn521	Carboxylic group		
		His551	Methoxy group		
		Tyr553	Hydroxyl group		
Compound (**4**)	−7.63	Arg372	Phenolic ring (cent.)	Glu381	Flavone ring (π)
		Ile604	Phenolic ring (cent.)	Ile402	Phenolic ring (term.)
		Met608	Flavone ring	Lys405	Flavone ring (π)
				Leu516	Flavone ring Phenolic ring (term.)
				Phe520	Flavone ring (π)
				Met608	Flavone rings (π)
Compound (**5**)	−4.41	Gln509	Carboxylic group	Cys489	Phenyl ring
		Asp513	Carboxylic group		
		Asn521	Carboxylic group		
		Ser511	Hydroxyl group		
		Tyr553	Hydroxyl group		
Compound (**6**)	−8.74	Phe374	Flavone ring	Lys371	Flavone ring (π)
		Arg603	Sugar moiety	Phe374	Flavone ring
		Met606	Sugar moiety	Ile402	Phenolic ring
				Leu516	Flavone ring
				Phe520	Flavone ring (π)
				Arg603	Flavone ring (π)
				Met608	Phenolic ring (π)

**Table 2:** Docking results, binding modes and interactions of pure compounds isolated from *Z. floridana n*-BuOH fraction in the active site of CDK-2 (PDB ID: 1FVT).

Compound	Docking Score (kcal/mol)	H-bond Interaction		Hydrophobic Interaction	
		Amino Acid	Fragment	Amino Acid	Fragment
106	−6.31	His84	SO_2_NH_2_	Ile10	Phenyl ring
		Asp86	SO_2_NH_2_	Val18	Indole ring
		Asp145	NH (Indole ring)	Ala31	Br group
				Val64	Br group
				Leu134	Br group
				Ala144	Indole ring
Compound (**1**)	−7.62	Asp92	Flavone ring	Val18	Phenolic ring Methoxy group
		Lys129	Flavone ring	Phe80	Methoxy group
				Lys88	Flavone ring
				Lys89	Flavone ring
				Ala144	Phenyl ring Methoxy group
Compound (**2**)	−7.58	Lys88	Flavone ring	Val18	Phenolic ring Methoxy group
		Lys89	Flavone ring	Lys88	Flavone ring
		Asp92	Flavone ring	Lys89	Flavone ring
		Lys129	Flavone ring	Ala144	Phenolic ring
Compound (**3**)	−5.01	Leu83	Hydroxyl group	Ile10	Phenyl ring Methoxy group
				Val18	Phenyl ring
				Ala31	Phenyl ring Methoxy group
				Val64	Methoxy group
				Leu134	Phenyl ring Methoxy group
Compound (**4**)	−7.60	His84	Hydroxyl group	Ile10	Phenolic ring Flavone ring
		Lys129	Flavone ring	Val18	Phenolic rings
				Asp86	Flavone ring
				Lys89	Flavone ring
				Ala144	Phenolic ring
Compound (**5**)	−4.50	Glu81	Hydroxyl group	Ala31	Phenyl ring
		Leu83	Hydroxyl group	Val18	Phenyl ring
				Leu134	Phenyl ring
Compound (**6**)	−8.38	His84	Sugar moiety	Ile10	Flavone ring
		Asp86	Sugar moiety	Val18	Phenolic ring Flavone ring
		Lys89	Sugar moiety	Ala31	Phenolic ring
		Asp145	Phenolic ring	Phe80	Phenolic ring
				Leu134	Flavone ring
				Ala144	Phenolic ring

## Data Availability

All data are included in the main text and the supporting material file.

## Supplementary Materials

The following supporting information can be downloaded at: https://www.mdpi.com/article/10.3390/metabo13010010/s1, Table S1: Toxoplasmocidal effect of *Z. floridana* methanol extract and its different fractions against *T. gondii*; Table S2: The percent inhibition of the cancer cells’ viability under the effect of the different tested concentrations of *Z. floridana* leaves MeOH extracts; Table S3: The percent inhibition of the cancer cells’ viability under the effect of the different tested concentrations of *Z. floridana* leaves’ different fractions; Table S4: Cytotoxic effect of *Z. floridana* methanol extract against different cell lines; Table S5: Cytotoxic effect of *Z. floridana* different fractions against MCF-7, HCT-116 and WISH cell lines; Figure S1: Extraction and fractionation steps of *Z. floridana* leaves; Figure S2: Isolation steps of six pure compounds from *Z. floridana n*-BuOH fraction; Figure S3: UV spectrum of compound (**1**) in MeOH; Figure S4: IR spectrum of compound (**1**) in KBr disc; Figure S5: (A) ^1^H and (B) DEPTQ NMR spectrum of compound (**1**) (CD_3_OD); Figure S6: ESIMS “positive (A) and negative modes (B)” of compound (**1**); Figure S7: UV spectrum of compound (**2**) in MeOH; Figure S8: (A) IR spectrum of compound (**2**) in KBr disc (B) IR fingerprint spectrum of compound (**2**) and bilobetin authentic sample in KBr disc; Figure S9: (A) ^1^H and (B) APT NMR spectrum of compound (**2**) (CD_3_OD); Figure S10: ESIMS “positive (A) and negative modes (B)” of compound (**2**); Figure S11: UV spectrum of compound (**3**) in MeOH; Figure S12: (A) ^1^H and (B) APT NMR spectrum of compound (**3**) (CD_3_OD); Figure S13: ESIMS “positive (A) and negative modes (B)” of compound (**3**); Figure S14: UV spectrum of compound (**4**) in MeOH; Figure S15: (A) IR spectrum of compound (**4**) in KBr disc (B) IR fingerprint spectrum of compound (**4**) and Amentoflavone authentic sample in KBr disc; Figure S16: (A) ^1^H and (B) APT NMR spectrum of compound (**4**) (CD_3_OD); Figure S17: ESIMS “positive (A) and negative modes (B)” of compound (**4**); Figure S18: UV spectrum of compound (**5**) in MeOH; Figure S19: IR spectrum of compound (**5**) in KBr disc; Figure S20: (A) ^1^H and (B) APT NMR spectrum of compound (**5**) (CD_3_OD); Figure S21: ESIMS “positive (A) and negative modes (B)” of compound (**5**); Figure S22: UV spectrum of compound (**6**) in MeOH; Figure S23: IR spectrum of compound (**6**) in KBr disc; Figure S24: (A) ^1^H and (B) APT NMR spectrum of compound (**6**) (CD_3_OD); Figure S25: (A) HSQC and (B) HMBC spectrums of compound (**6**) (CD_3_OD); Figure S26: ESIMS “positive (A) and negative modes (B)” of compound (**6**); Figure S27: 2D (A) and 3D (B) patterns demonstrating the binding interaction of 1UE into active site of TS-DHFR (PDB code: 4KY4); Figure S28: 2D (A) and 3D (B) patterns demonstrating the binding interaction of compound (**1**) into active site of TS-DHFR (PDB code: 4KY4); Figure S29: 2D (A) and 3D (B) patterns demonstrating the binding interaction of compound (**2**) into active site of TS-DHFR (PDB code: 4KY4); Figure S30: 2D (A) and 3D (B) patterns demonstrating the binding interaction of compound (**3**) into active site of TS-DHFR (PDB code: 4KY4); Figure S31: 2D (A) and 3D (B) patterns demonstrating the binding interaction of compound (**4**) into active site of TS-DHFR (PDB code: 4KY4); Figure S32: 2D (A) and 3D (B) patterns demonstrating the binding interaction of compound (**5**) into active site of TS-DHFR (PDB code: 4KY4); Figure S33: 2D (A) and 3D (B) patterns demonstrating the binding interaction of compound (**6**) into active site of TS-DHFR (PDB code: 4KY4); Figure S34: 2D (A) and 3D (B) patterns demonstrating the binding interaction of (**106**) into active site of CDK-2 (PDB ID: 1FVT); Figure S35: 2D (A) and 3D (B) patterns demonstrating the binding interaction of compound **(1)** into active site of CDK-2 (PDB ID: 1FVT); Figure S36: 2D (A) and 3D (B) patterns demonstrating the binding interaction of compound (**2**) into active site of CDK-2 (PDB ID: 1FVT); Figure S37: 2D (A) and 3D (B) patterns demonstrating the binding interaction of compound (**3**) into active site of CDK-2 (PDB ID: 1FVT); Figure S38: 2D (A) and 3D (B) patterns demonstrating the binding interaction of compound (**4**) into active site of CDK-2 (PDB ID: 1FVT); Figure S39: 2D (A) and 3D (B) patterns demonstrating the binding interaction of compound (**5**) into active site of CDK-2 (PDB ID: 1FVT); Figure S40: 2D (A) and 3D (B) patterns demonstrating the binding interaction of compound (**6**) into active site of CDK-2 (PDB ID: 1FVT).
