# Supplementary material for: Evaluation of Zamia floridana A. DC. Leaves and Its Isolated Secondary Metabolites as Natural Anti-Toxoplasma and Anti-Cancer Agents Using In Vitro and In Silico Studies
Source: Metabolites. 2022 Dec 21;13(1):10. doi: 10.3390/metabo13010010 (PMC9866161; doi:10.3390/metabo13010010)
Supplement: Supplementary file 1 [file metabolites-13-00010-s001.zip › metabolites-2076770-supplementary.pdf]

metabolites

MDPI

Article

Evaluation of *Zamia floridana* A. DC. Leaves and Its Isolated Secondary Metabolites as Natural Anti-toxoplasma and Anti-cancer Agents Using In Vitro and In Silico Studies

Hosam M. El-Seadawy [1], Kamilia A. Abo El-Seoud [1], Mona El-Aasr [1], Haytham O. Tawfik [2], Wagdy Eldehna [3, 4, *] and Amany E. Ragab [1,*]

[1] Department of Pharmacognosy, Faculty of Pharmacy, Tanta University, Tanta 31527, Egypt;
[2] Department of Pharmaceutical Chemistry, Faculty of Pharmacy, Tanta University, Tanta 31527, Egypt
[3] Department of Pharmaceutical Chemistry, Faculty of Pharmacy, Kafrelsheikh University, Kafrelsheikh 33516, Egypt
[4] School of Biotechnology, Badr University in Cairo, Badr City 11829, Egypt
* Correspondence: amany.ragab@pharm.tanta.edu.eg (A.E.R.); wagdy2000@gmail.com (W.E.)

Supplementary Materials

Table S1. Toxoplasmocidal effect of *Z. floridana* methanol extract and its different fractions.

Drug	EC_{50} ± SEM (µg/mL)
Cotrimoxazole	4.18±0.3
Z. floridana MeOH extract	8.19±0.4
Pet. ether fraction	31.95±1.3
Chloroform fraction	16.71±0.8
Ethyl acetate fraction	9.74±0.5
n-butanol fraction	7.16±0.4

Table S2. The percent inhibition of the cancer cells' viability under the effect of the different tested concentrations of *Z. floridana* leaves MeOH extracts.

Conc. (µg/mL)	HEPG-2	MCF-7	HCT-116	PC3	HELA	WISH
			**Doxorubicin**			
**100**	6.3	6.2	7.1	8.8	7.3	8.4
**50**	11.2	10.9	13.9	16.3	12.1	17.1
**25**	14.1	14.3	18.7	21.7	18.9	24.3
**12.5**	28.3	26.9	31.4	38.9	30.8	33.5
**6.25**	45.8	41.5	47.9	59.2	51.7	56.6
**3.125**	57.6	58.4	60.5	73.6	62.4	69.2
**1.56**	71.2	69.1	73.8	95.3	74.0	88.6
			***Z. floridana* MeOH extract**			
**100**	26.8	20.1	27.3	35.7	33.1	31.2
**50**	40.2	31.7	38.1	50.5	42.7	47.3
**25**	52.1	45.2	47.2	62.6	54.2	54.1
**12.5**	68.7	52.8	65.0	71.3	66.9	76.2
**6.25**	84.9	76.3	78.4	92.8	87.0	88.4
**3.125**	99.4	95.2	91.8	100	100	100
**1.56**	100	100	100	100	100	100

Table S3. The percent inhibition of the cancer cells' viability under the effect of the different tested concentrations of *Z. floridana* leaves' different fractions.

Conc. (μg/ml)	MCF-7	HCT-116	WISH
**PE fraction**			
**100**	34.8	31.6	49.9
**50**	47.3	41.9	75.2
**25**	61.2	54.4	81.1
**12.5**	73.6	65.1	89.9
**6.25**	88.1	79.6	99.4
**3.125**	100	98.2	100
**1.56**	100	100	100
**$CHCl_3$ fraction**			
**100**	28.7	27.8	48.9
**50**	41.0	38.2	66.2
**25**	57.2	49.3	75.7
**12.5**	61.4	63.7	91.4
**6.25**	80.5	78.6	98.1
**3.125**	98.9	95.1	100
**1.56**	100	100	100
**EtOAc fraction**			
**100**	23.8	7.9	34.6
**50**	34.9	16.3	52.5
**25**	48.0	27.0	60.0
**12.5**	54.7	38.2	84.5
**6.25**	75.2	54.1	97.1
**3.125**	93.1	78.4	100
**1.56**	100	95.9	100
***n*-BuOH fraction**			
**100**	5	21.3	37.4
**50**	26.3	28.2	56.7
**25**	35.6	37.8	64.9
**12.5**	40.9	51.4	89.9
**6.25**	62.4	75.1	97.2
**3.125**	81.2	93.9	100
**1.56**	96.1	100	100

Table S4. Cytotoxic effect of *Z. floridana* methanol extract against different cell lines.

**Drug**	***In vitro* Cytotoxicity IC_{50} ± SEM (μg/mL)**					
	HEPG-2	MCF-7	HCT-116	PC3	HELA	WISH
Doxorubicin	4.50±0.2	4.17±0.2	5.23±0.3	8.87±0.6	5.57±0.4	7.79±0.5
Total MeOH extract of *Z. floridana*	31.87±2.6	20.57±1.7	27.33±2.3	48.48±3.5	36.36±2.9	40.29±3.2

Table S5. Cytotoxic effect of *Z. floridana* different fractions against MCF-7, HCT-116 and WISH cell lines.

**Drug**	***In vitro* Cytotoxicity IC_{50} ± SEM (μg/mL)**		
	MCF-7	HCT-116	WISH
Doxorubicin	4.17±0.2	5.23±0.3	7.79±0.5
Pet. ether fraction	45.06±3.1	33.78±2.5	95.61±4.6
Chloroform fraction	32.39±2.3	28.16±2.1	84.15±4.4
Ethyl acetate fraction	22.89±1.8	9.04±0.8	54.26±3.1
n-Butanol fraction	12.33±1.1	17.88±1.4	59.44±3.2

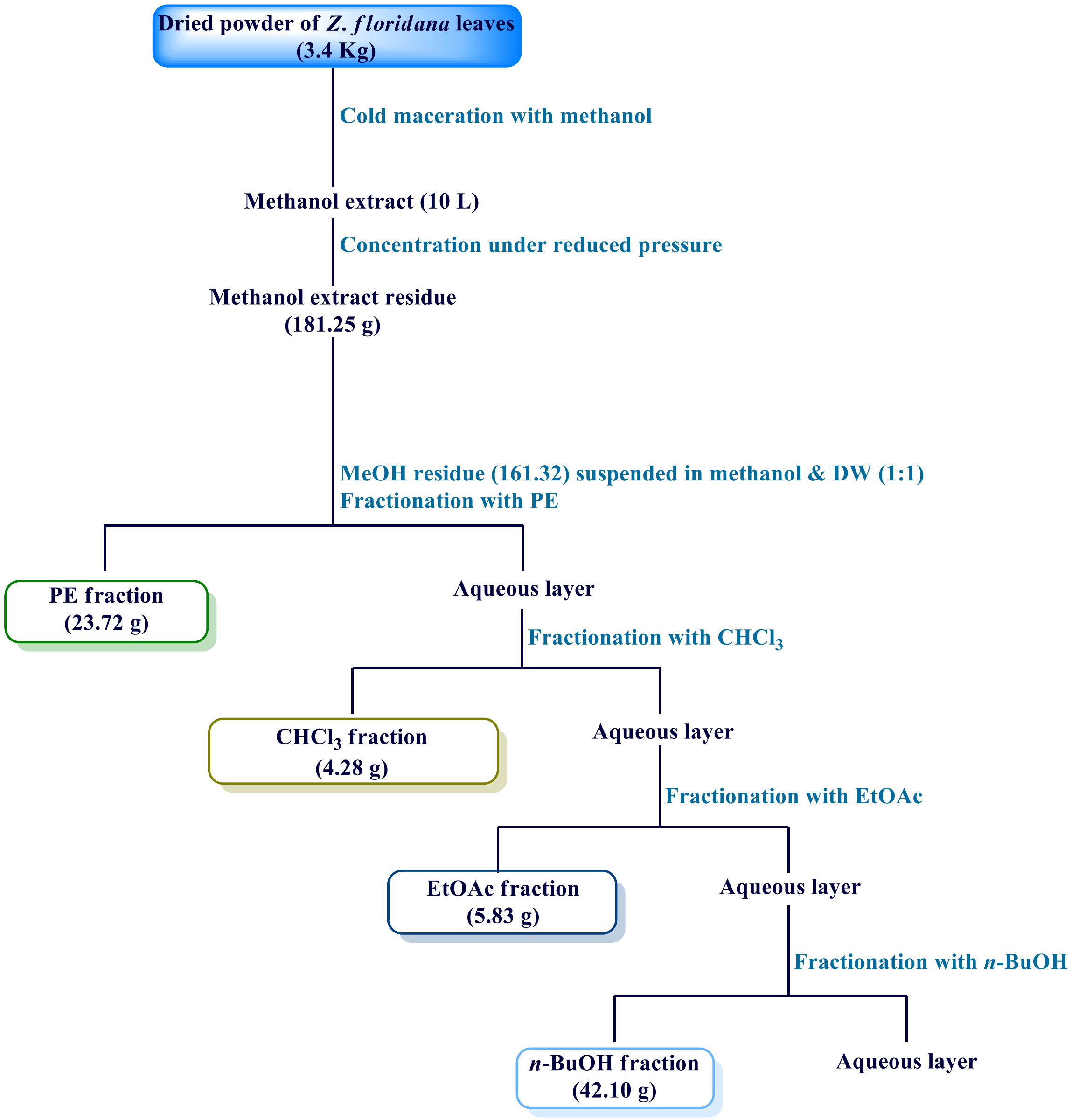

Figure S1. Extraction and fractionation steps of *Z. floridana* leaves

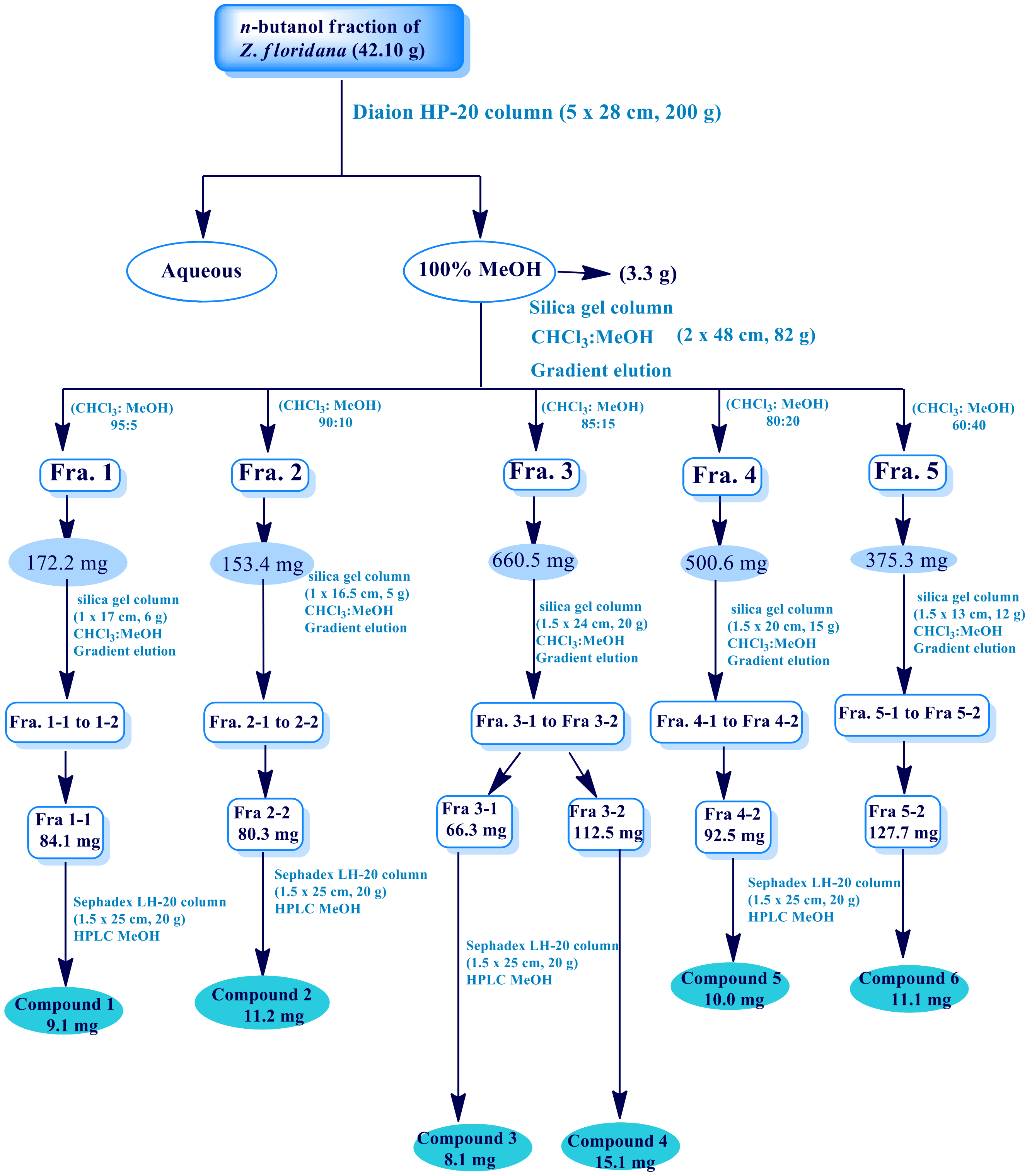

Figure S2. Isolation steps of six pure compounds from *Z. floridana n*-BuOH fraction

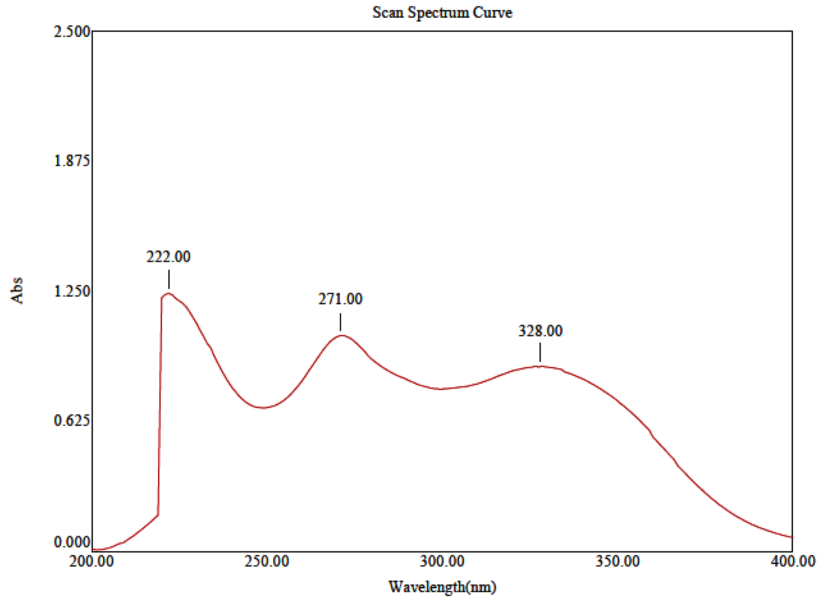

Figure S3. UV spectrum of compound (**1**) in MeOH

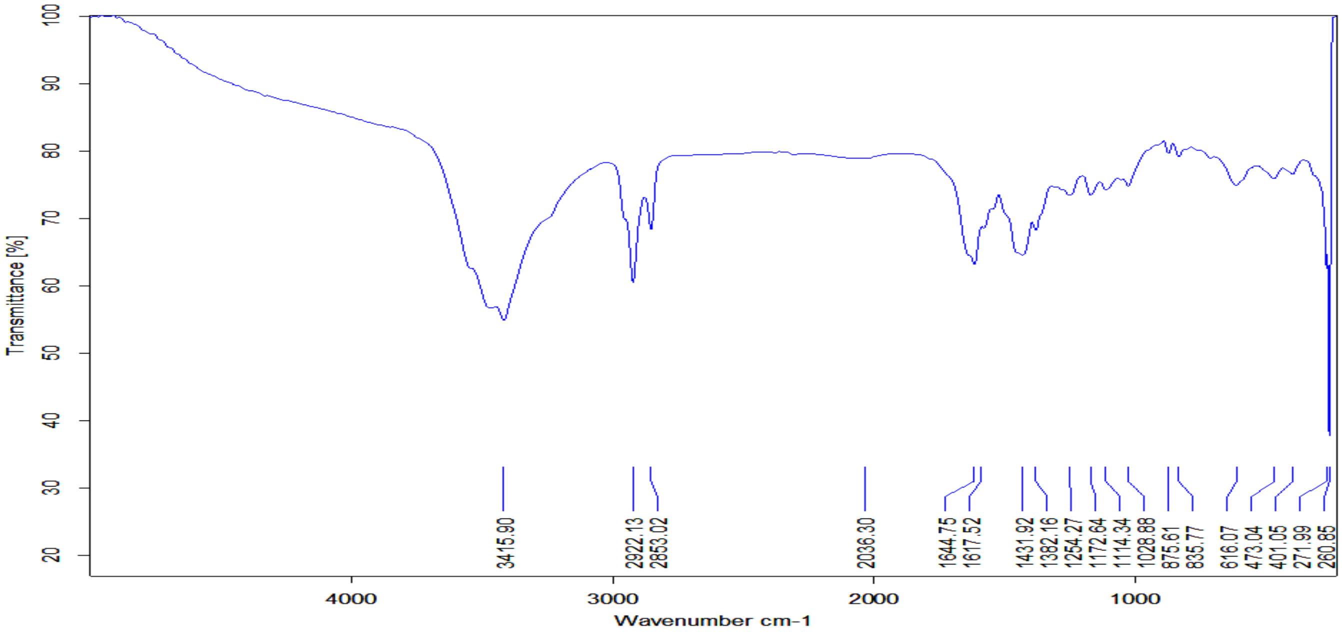

Figure S4. IR spectrum of compound (**1**) in KBr disc

(A)

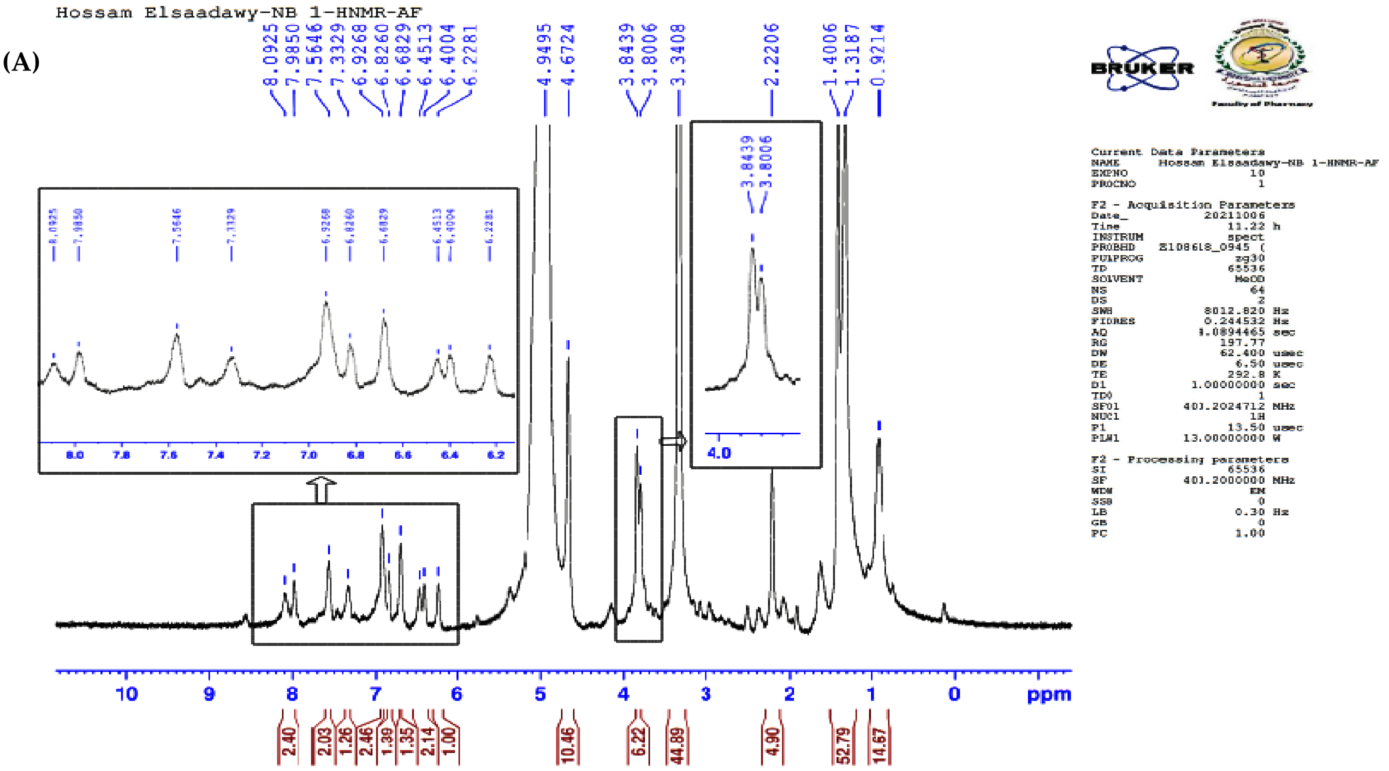

(B)

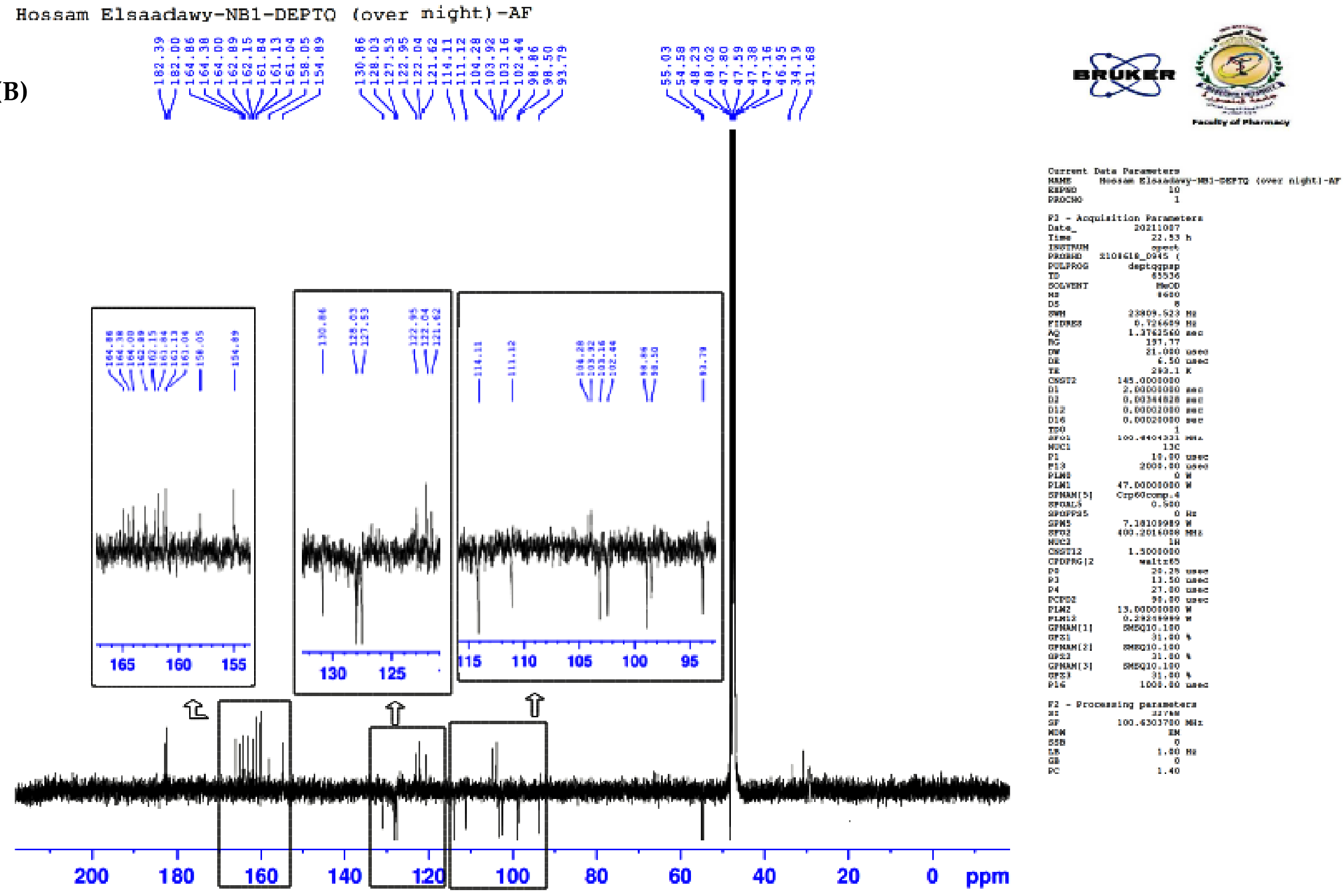

Figure S5. (A) ^{1}H and (B) DEPTQ NMR spectrum of compound (**1**) (CD_3OD)

(A)

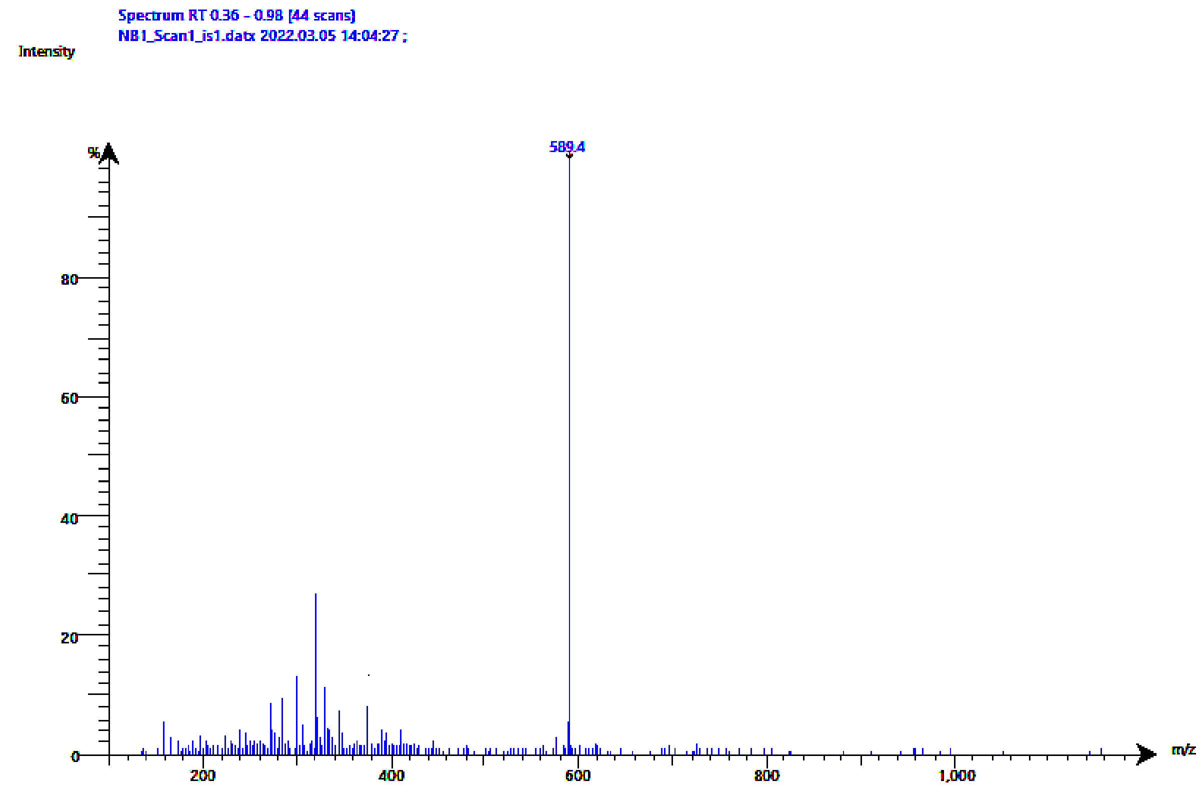

(B)

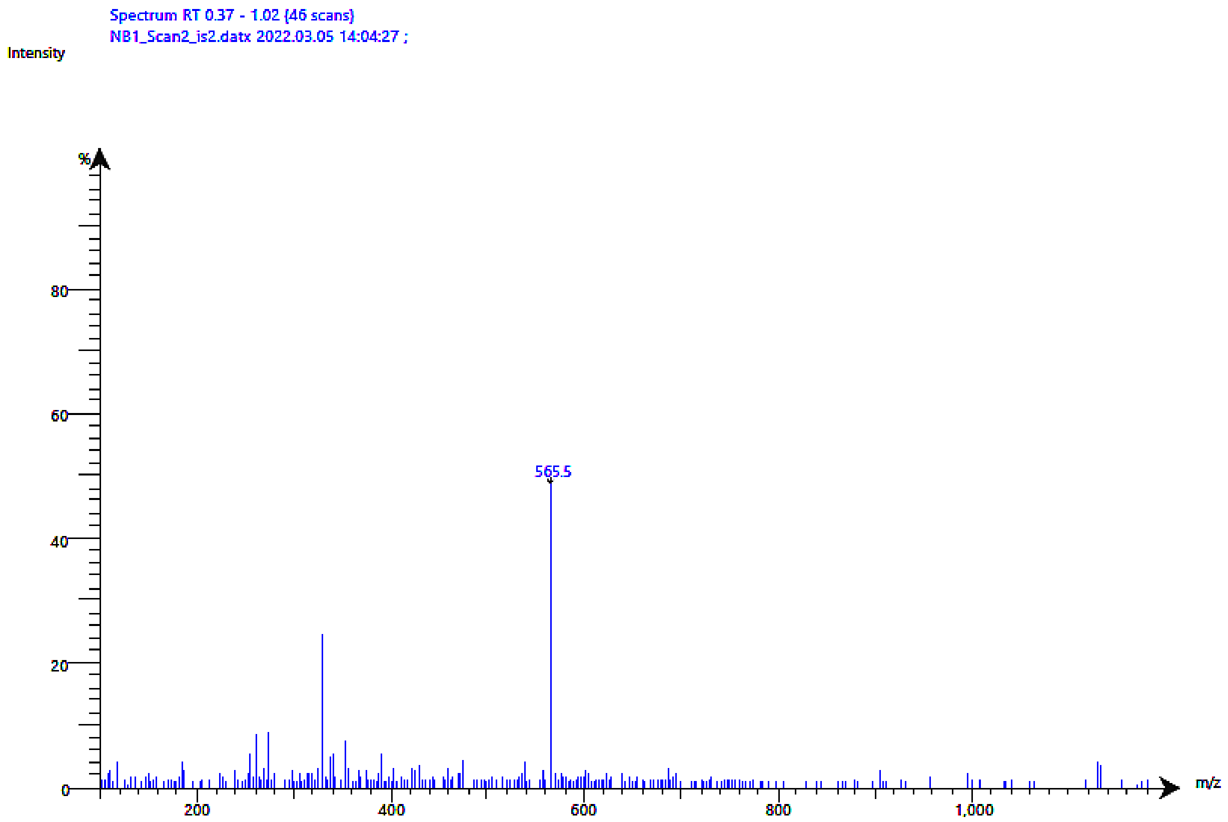

Figure S6. ESIMS "positive (A) and negative modes (B)" of compound (**1**)

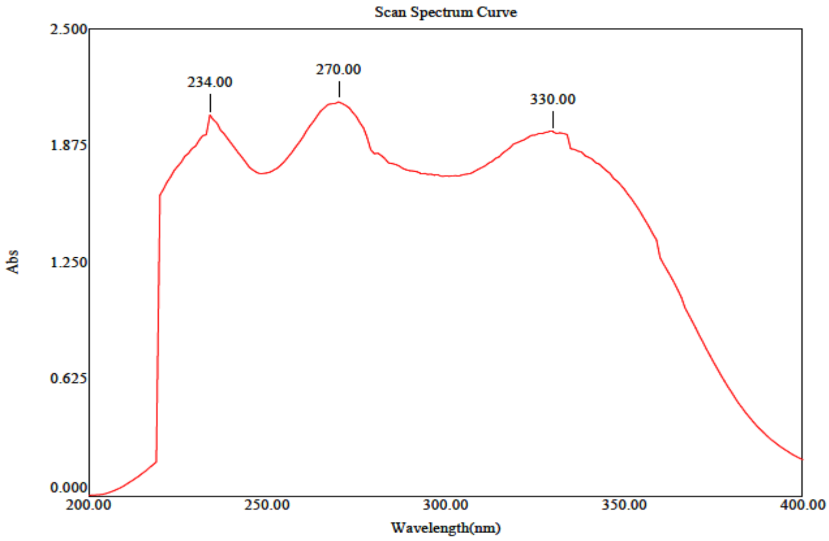

Figure S7. UV spectrum of compound (**2**) in MeOH

(A)

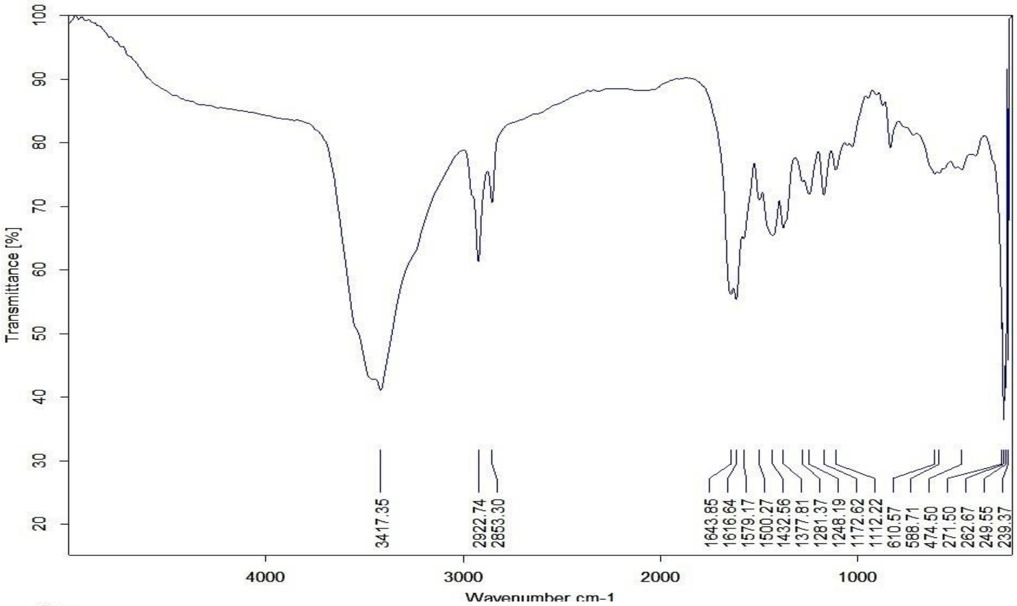

(B)

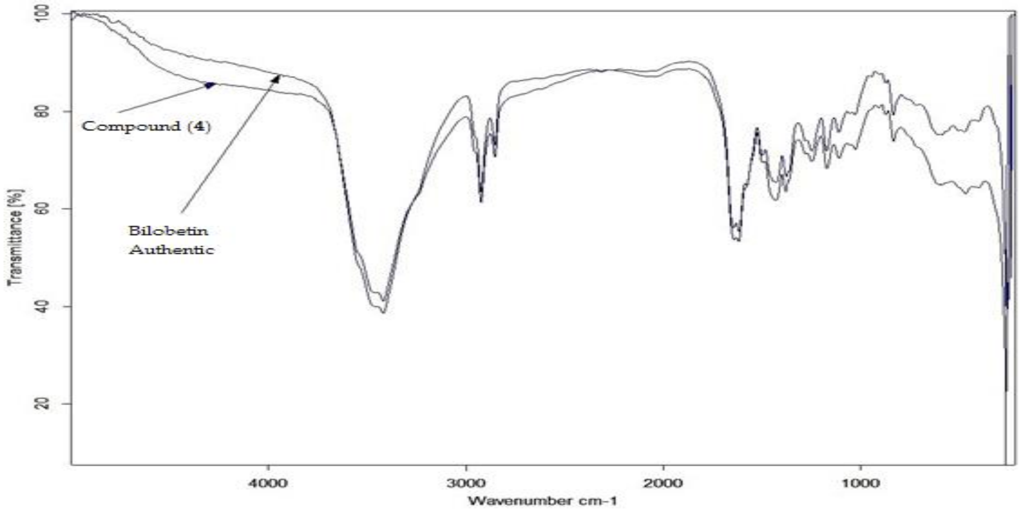

Figure S8. (A) IR spectrum of compound (**2**) in KBr disc

(B) IR fingerprint spectrum of compound (**2**) and bilobetin authentic sample in KBr

(A)

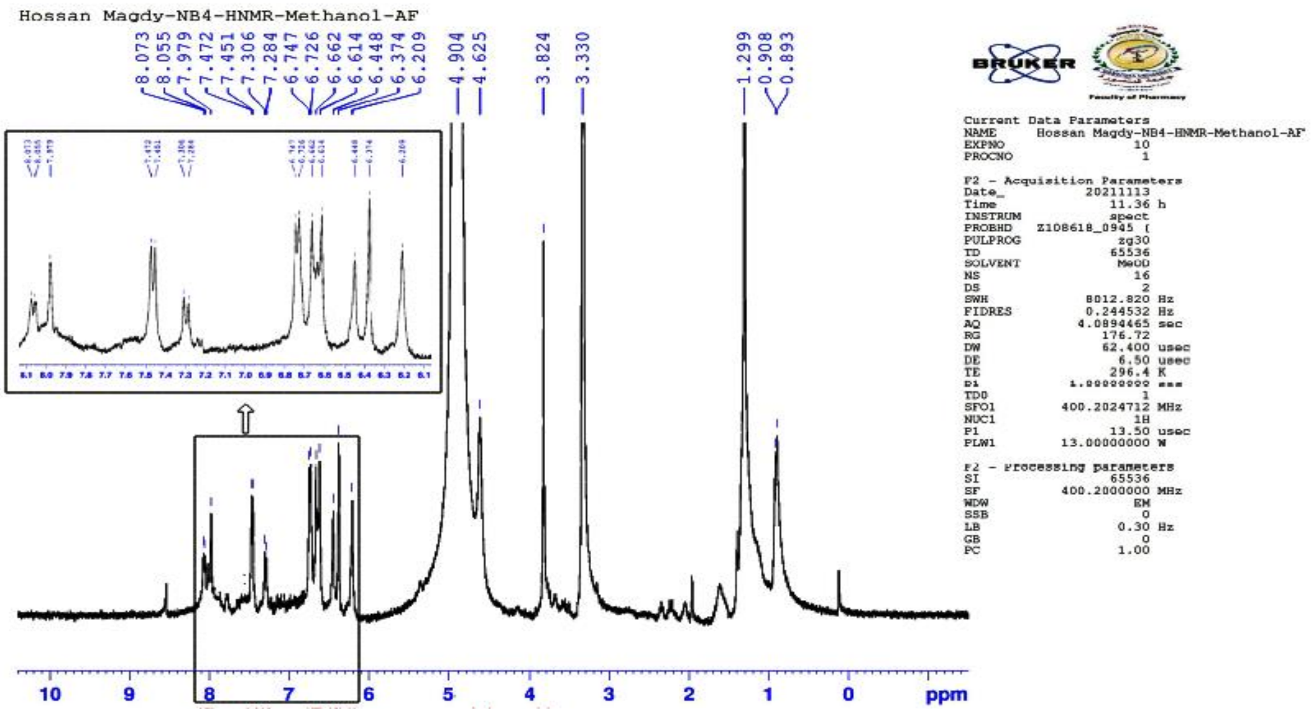

(B)

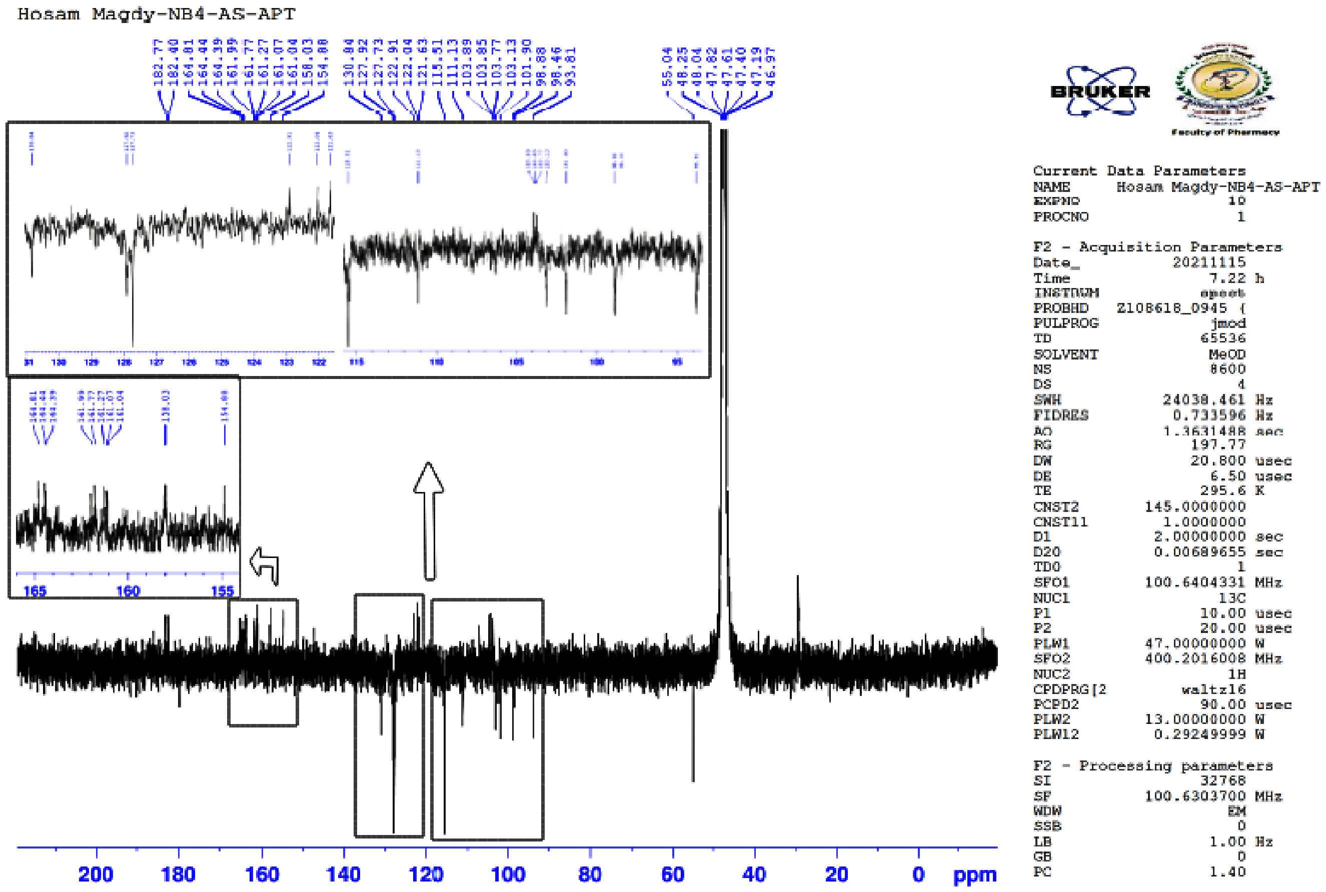

Figure S9. (A) ^{1}H and (B) APT NMR spectrum of compound (**2**) (CD_3OD)

(A)

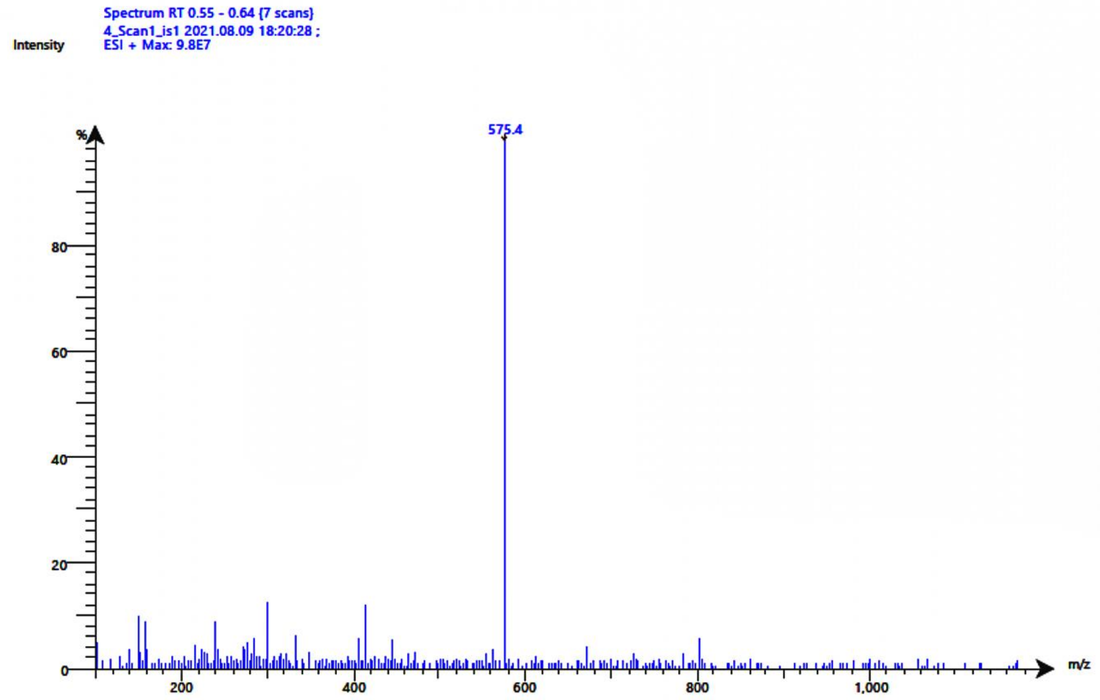

(B)

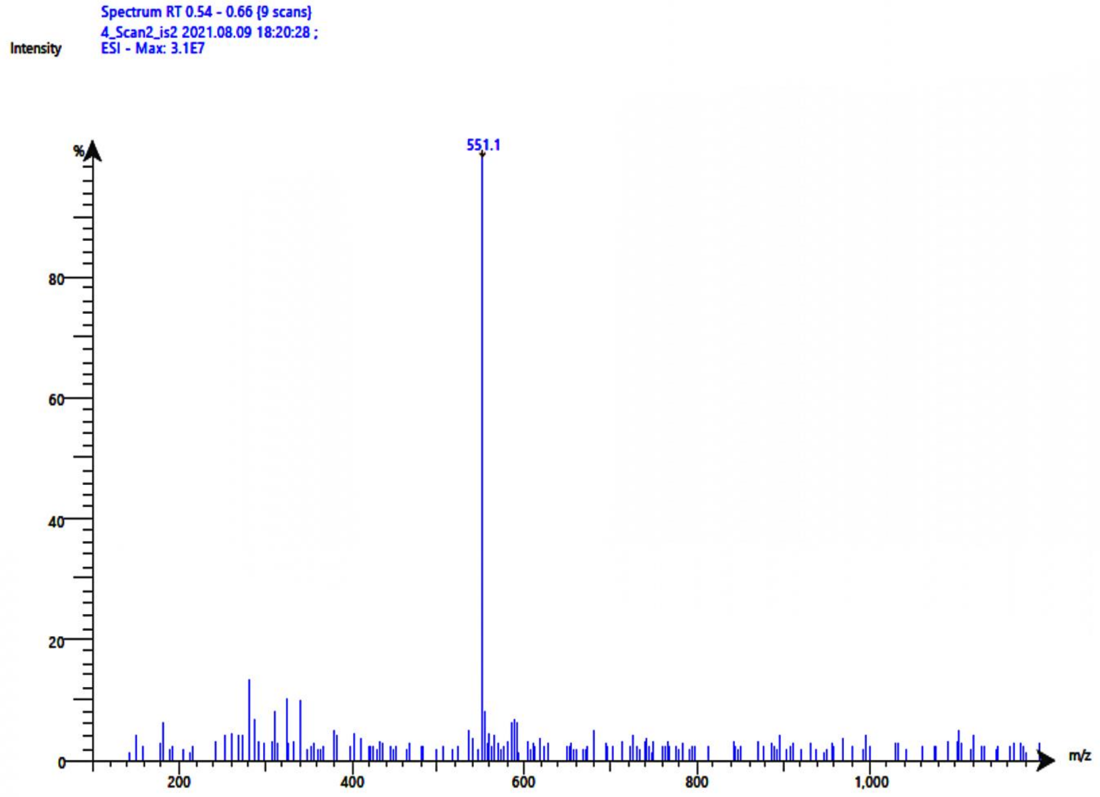

Figure S10. ESIMS "positive (A) and negative modes (B)" of compound (**2**)

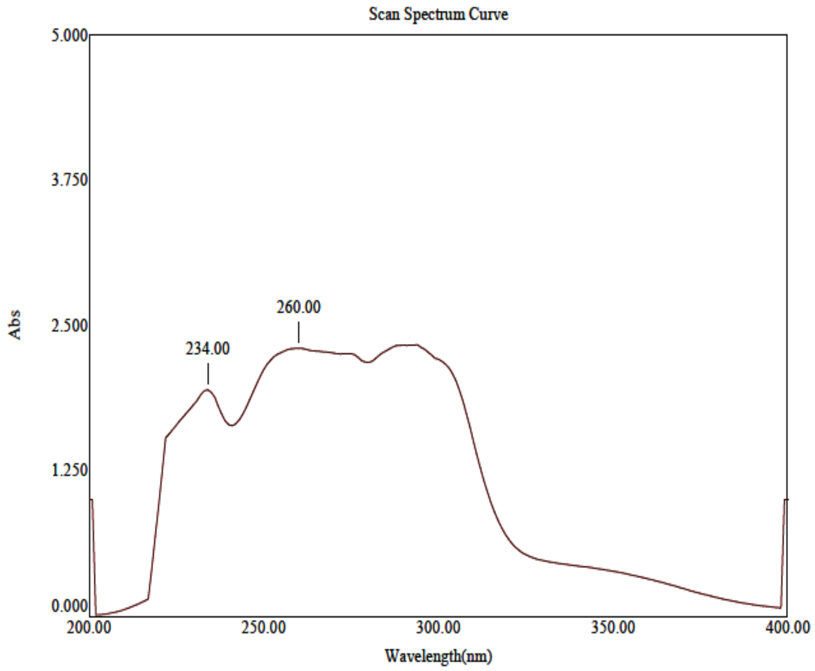

Figure S11. UV spectrum of compound (**3**) in MeOH

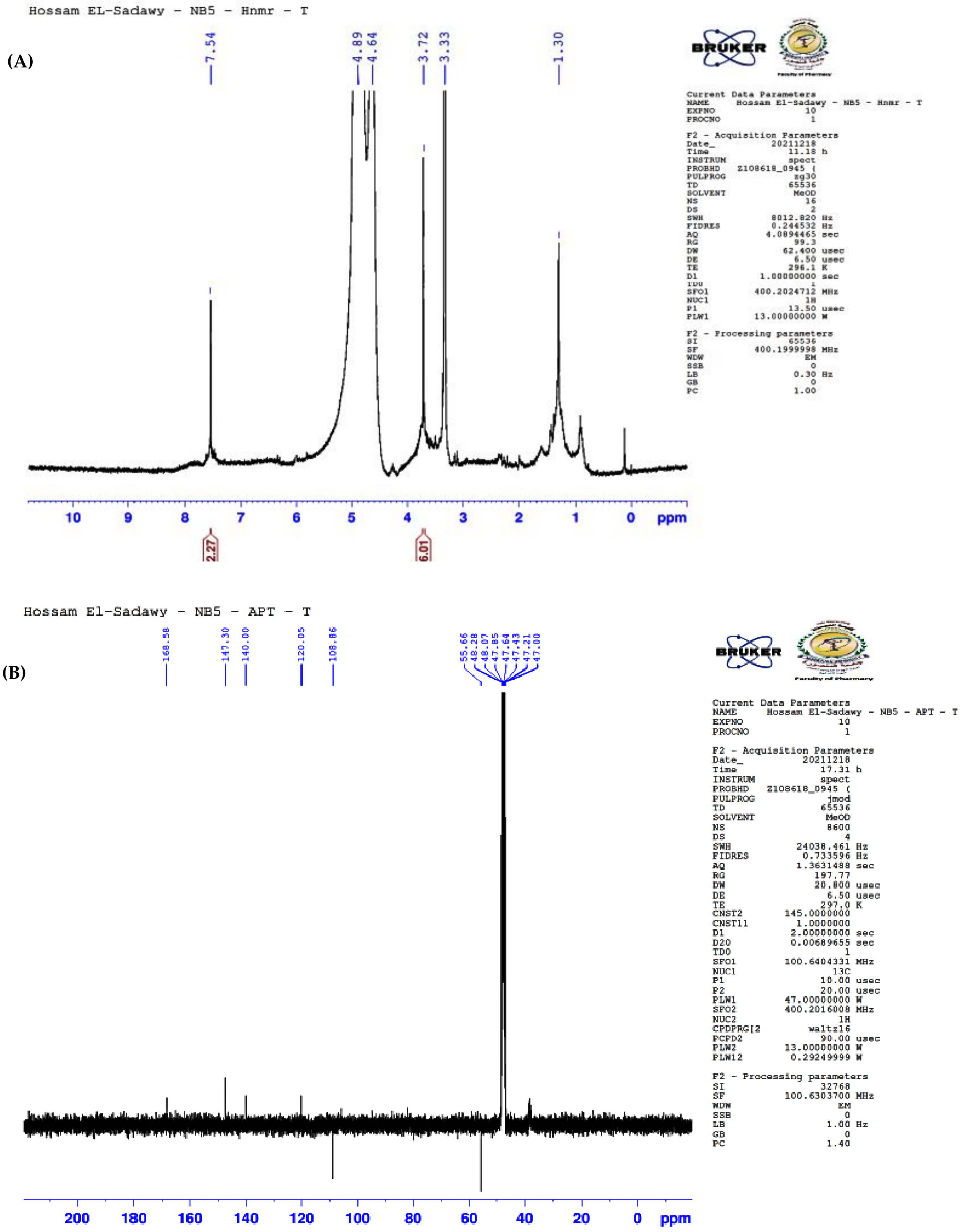

Figure S12. (A) ^{1}H and (B) APT NMR spectrum of compound (**3**) (CD_3OD)

(A)

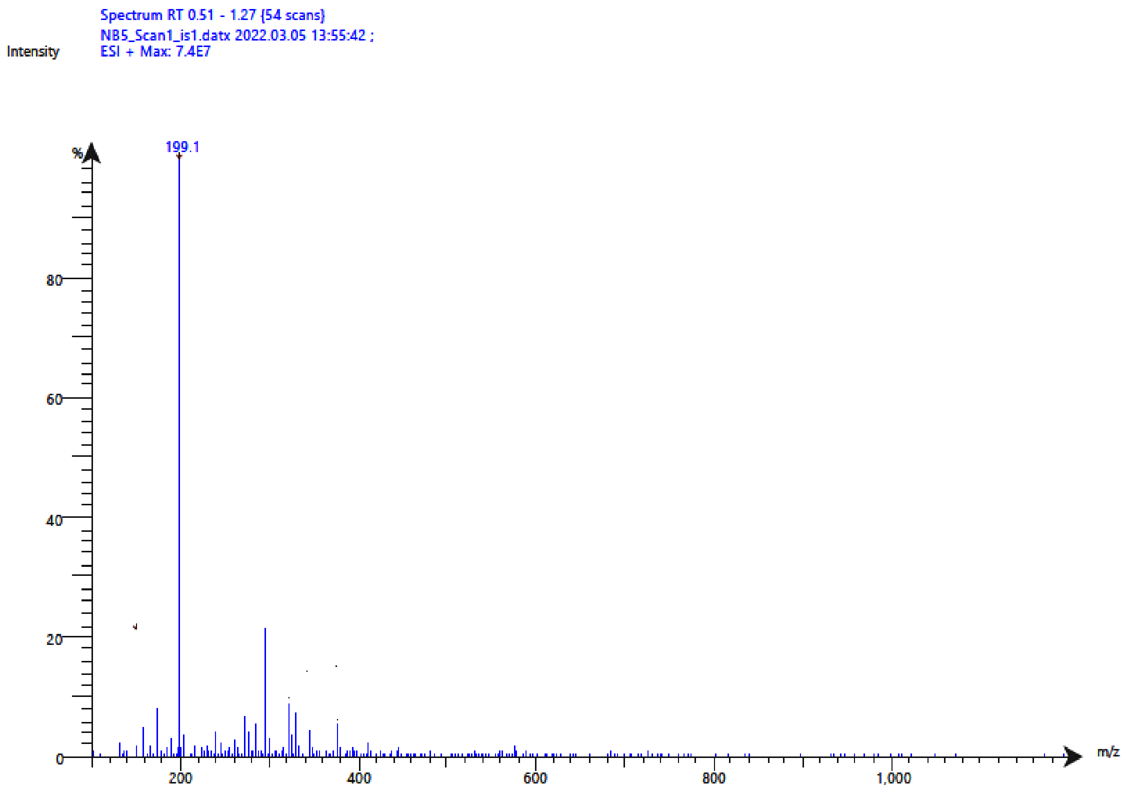

(B)

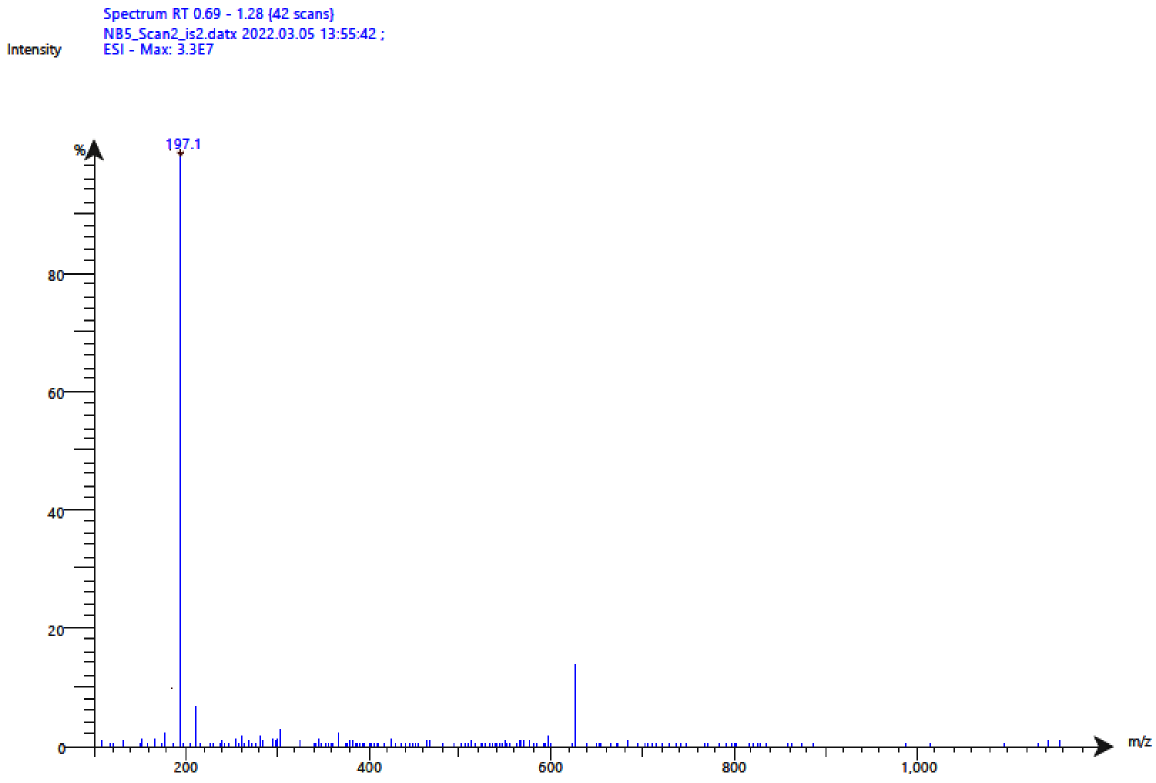

Figure S13. ESIMS "positive (A) and negative modes (B)" of compound (**3**)

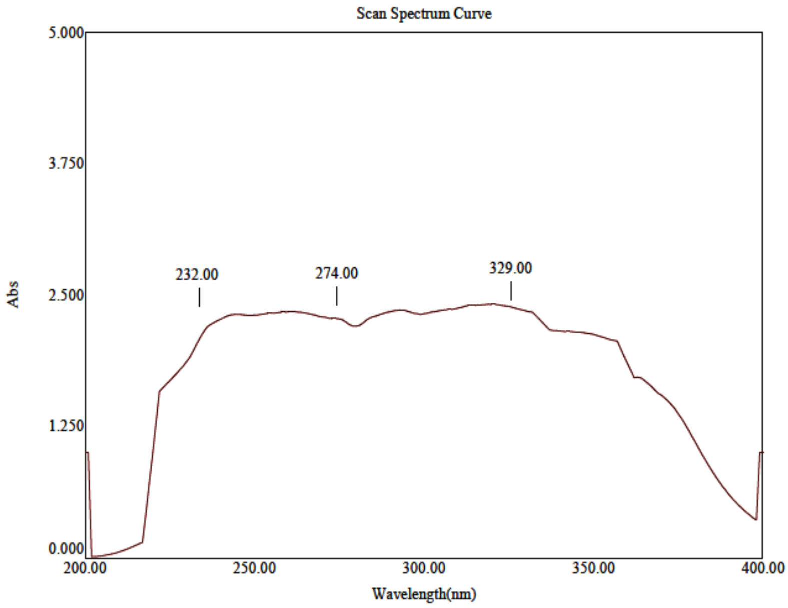

Figure S14. UV spectrum of compound (**4**) in MeOH

(A)

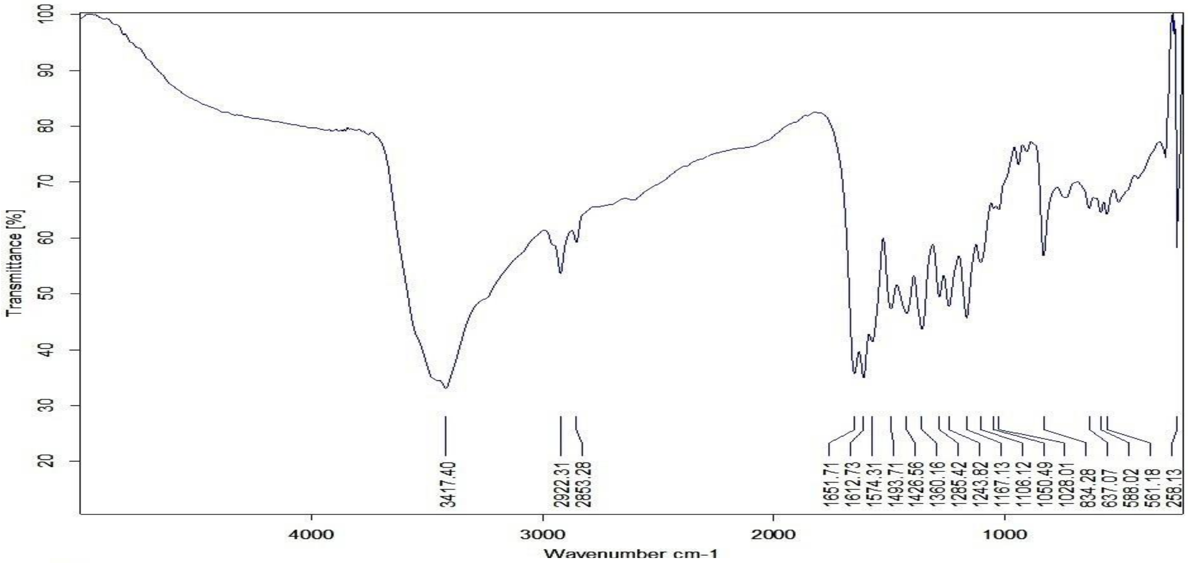

(B)

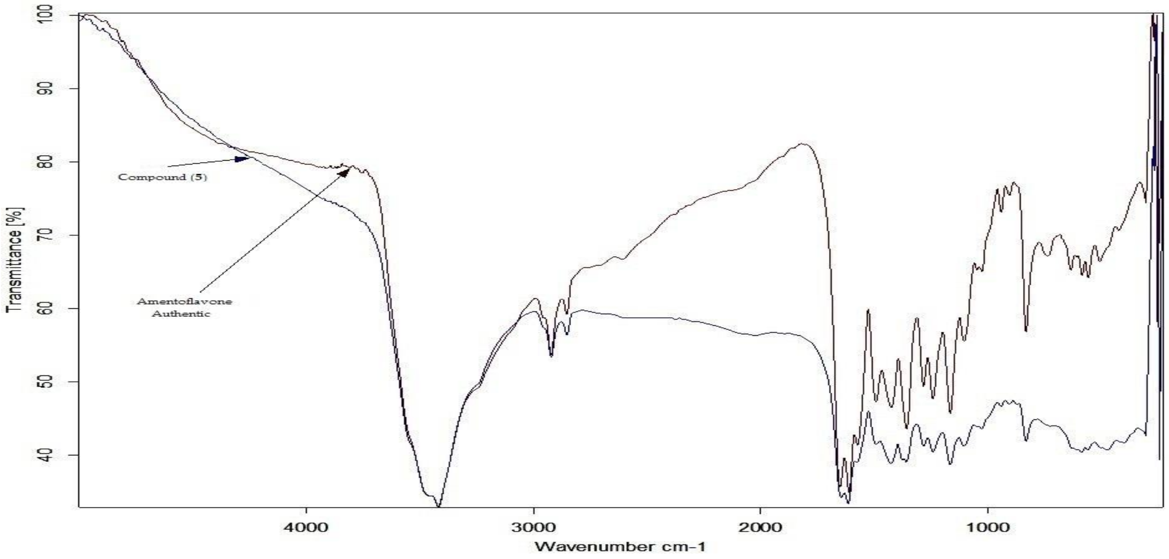

Figure S15. (A) IR spectrum of compound (**4**) in KBr disc

(B) IR fingerprint spectrum of compound (**4**) and Amentoflavone authentic sample in KBr disc

(A)

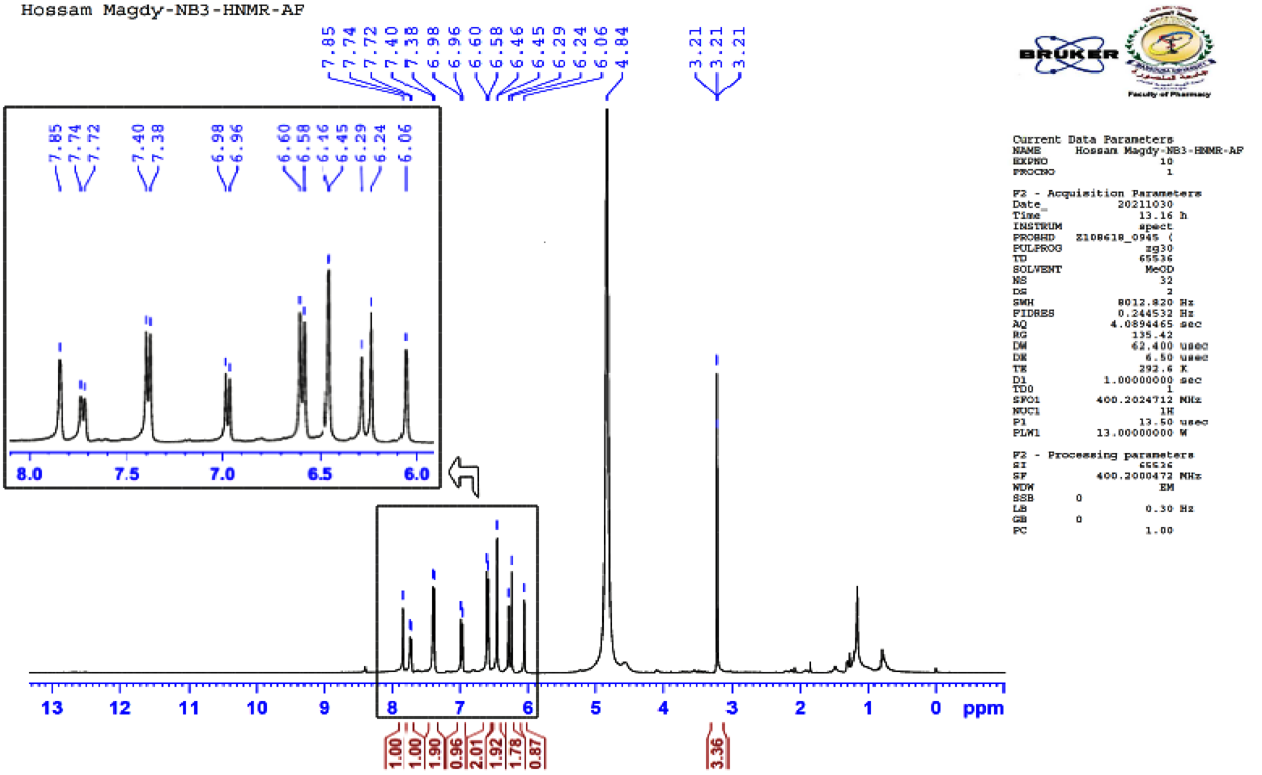

(B)

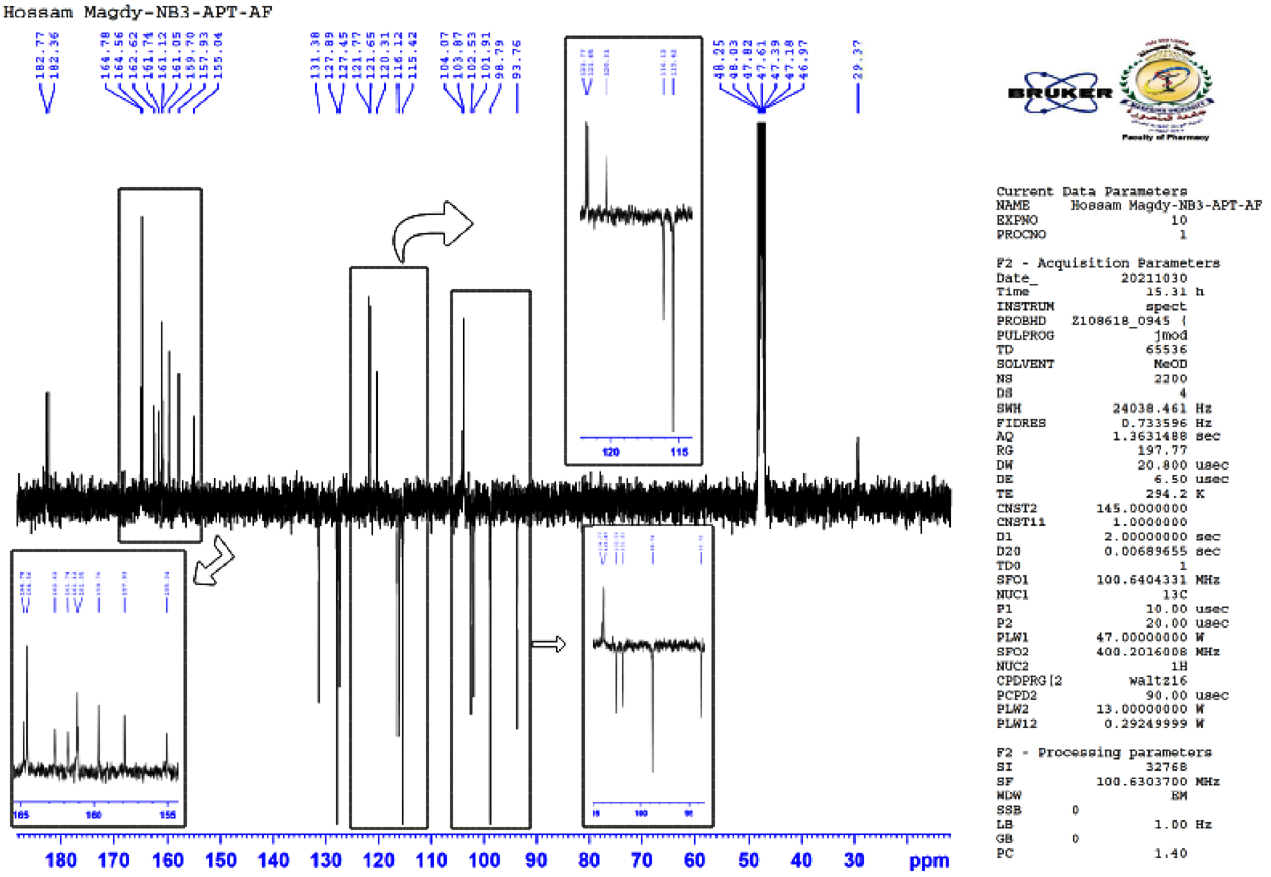

Figure S16. (A) ^{1}H and (B) APT NMR spectrum of compound (**4**) (CD_3OD)

(A)

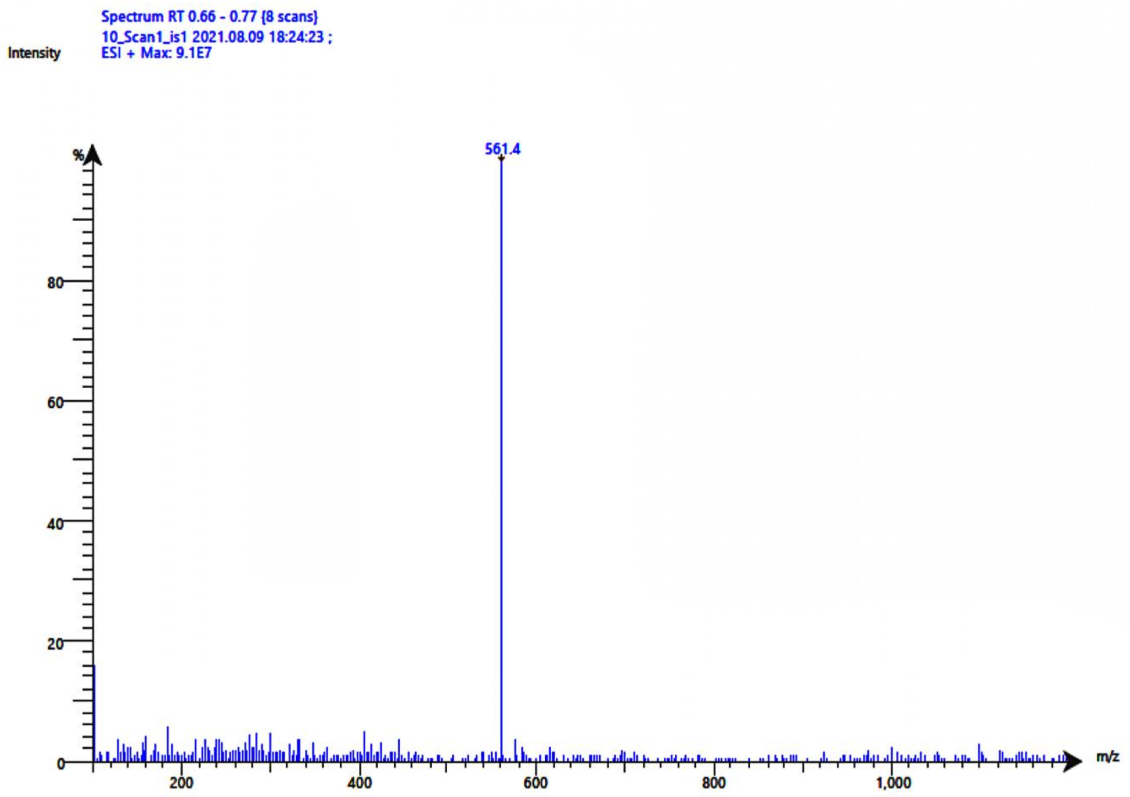

(B)

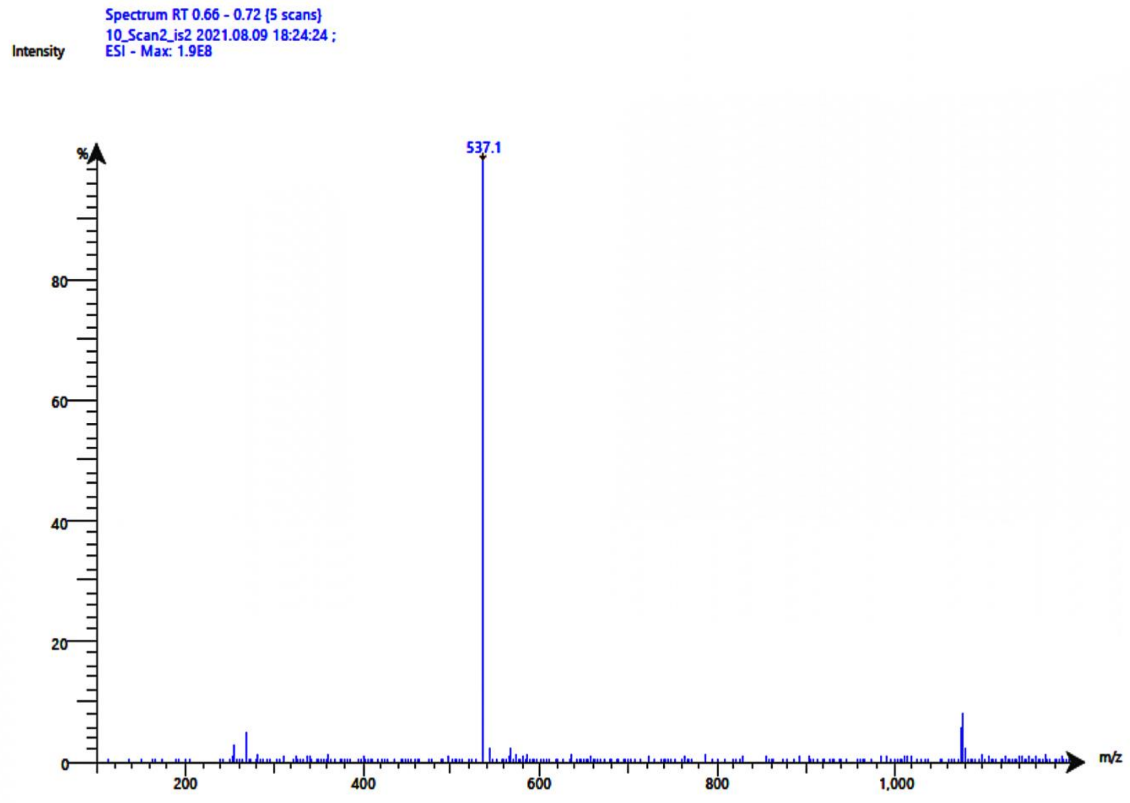

Figure S17. ESIMS ''positive (A) and negative modes (B)'' of compound (**4**)

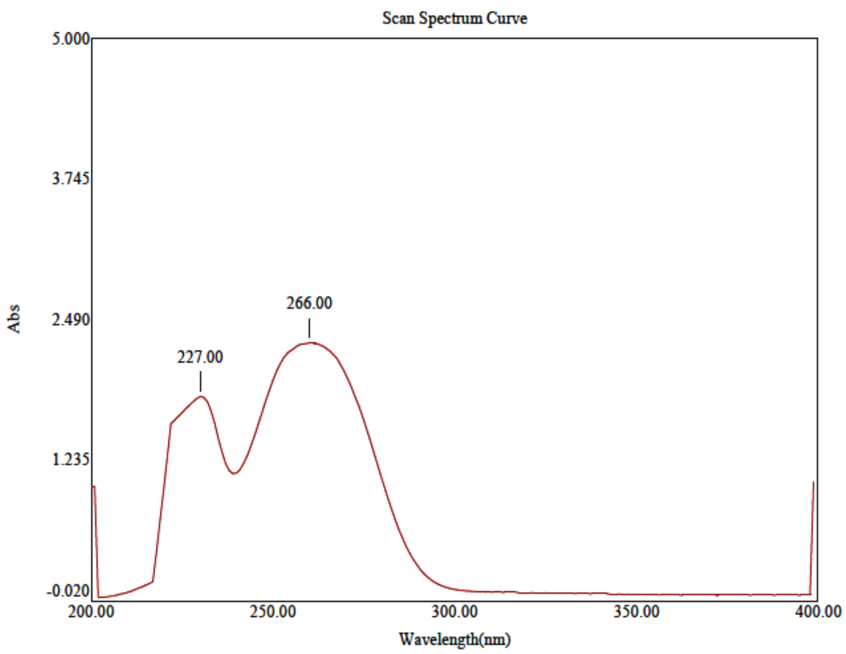

Figure S18. UV spectrum of compound (**5**) in MeOH

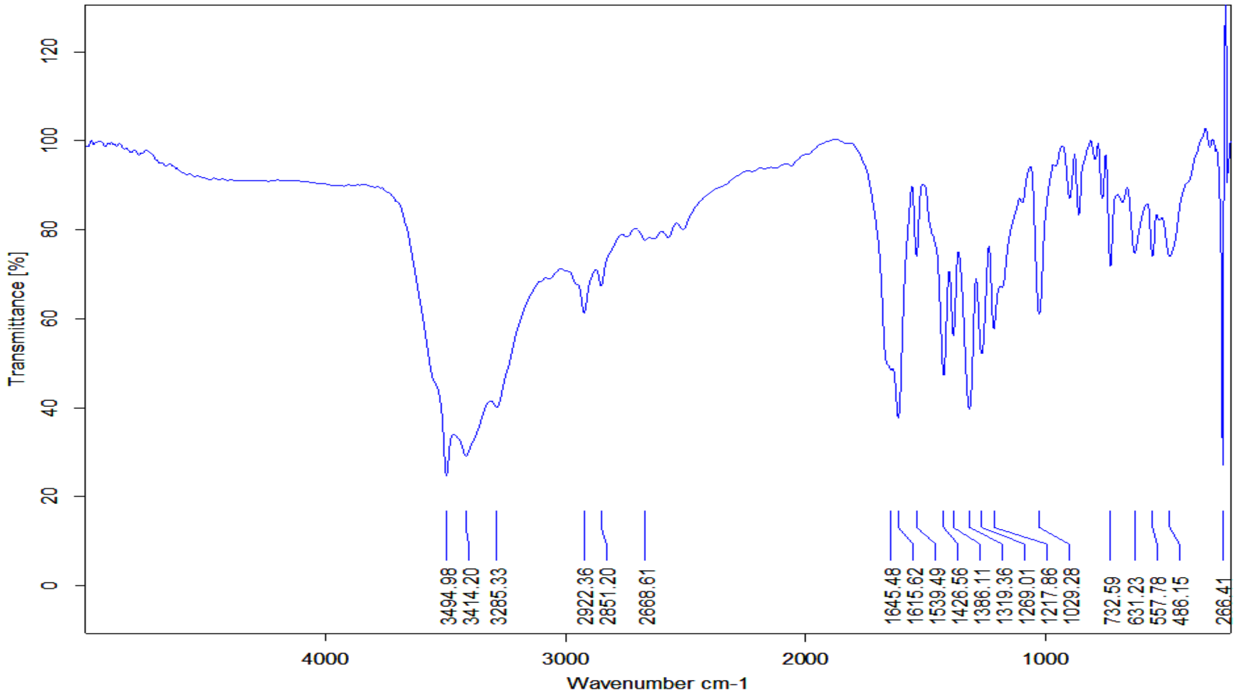

Figure S19. IR spectrum of compound (**5**) in KBr disc

(A)

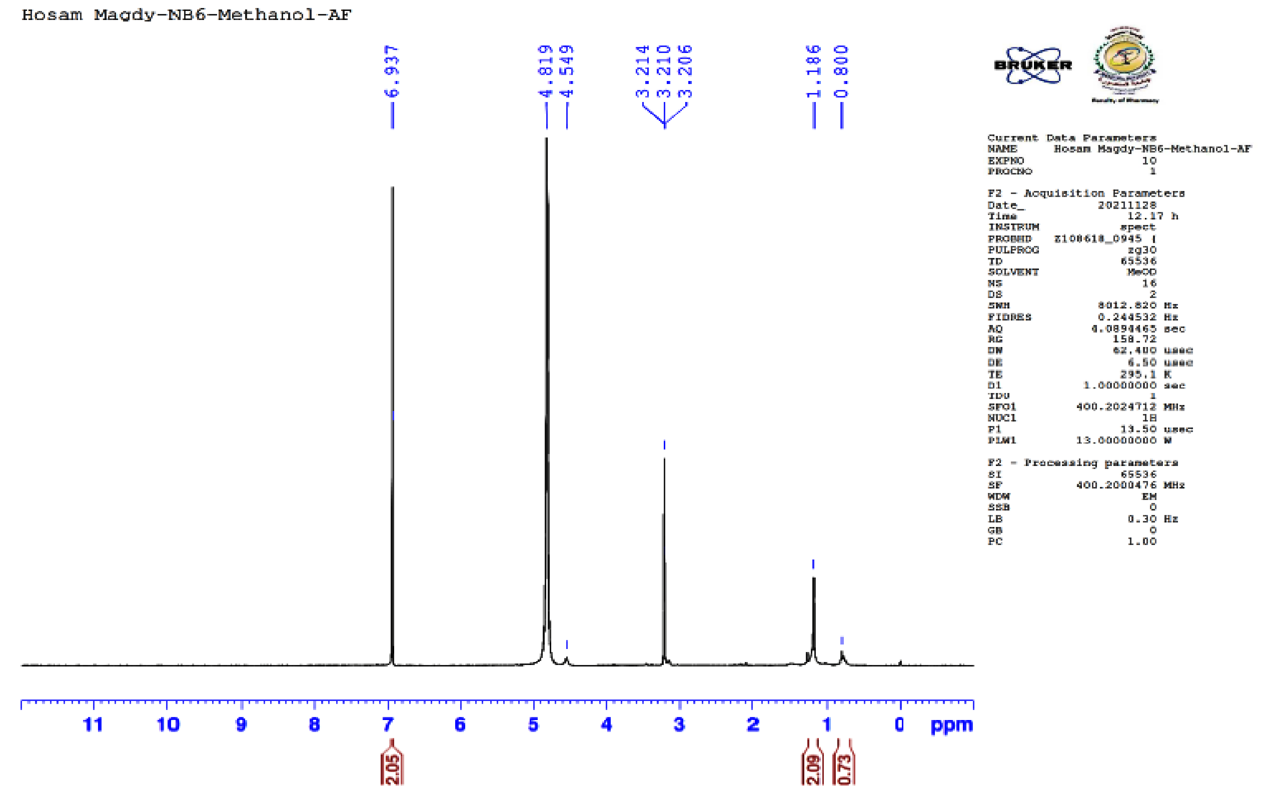

(B)

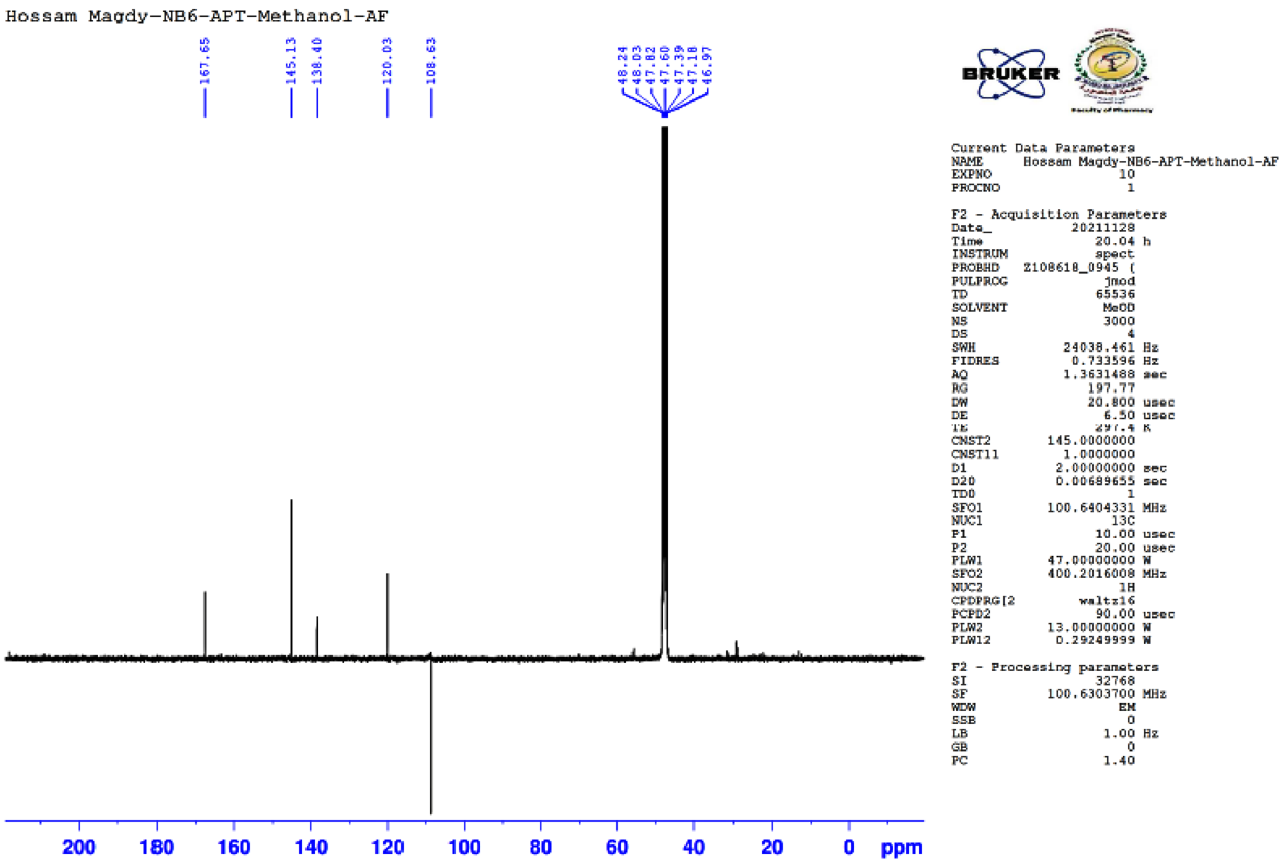

Figure S20. (A) ^{1}H and (B) APT NMR spectrum of compound (**5**) (CD_3OD)

(A)

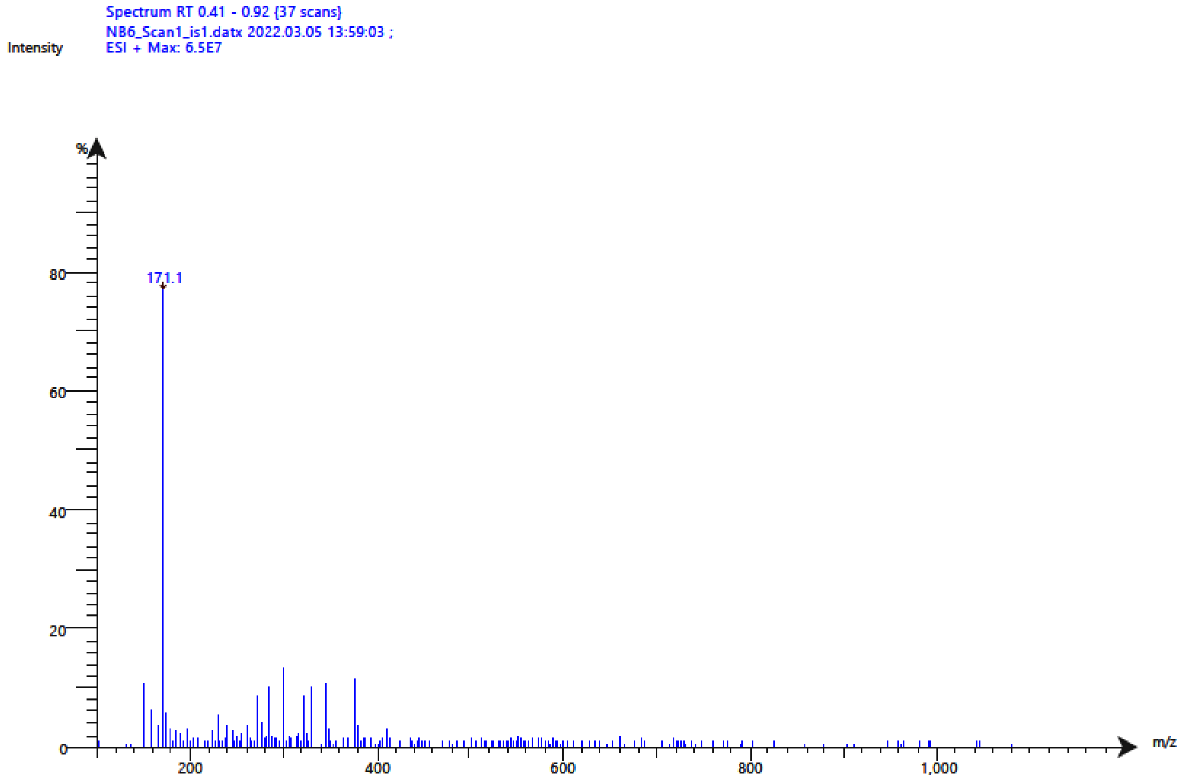

(B)

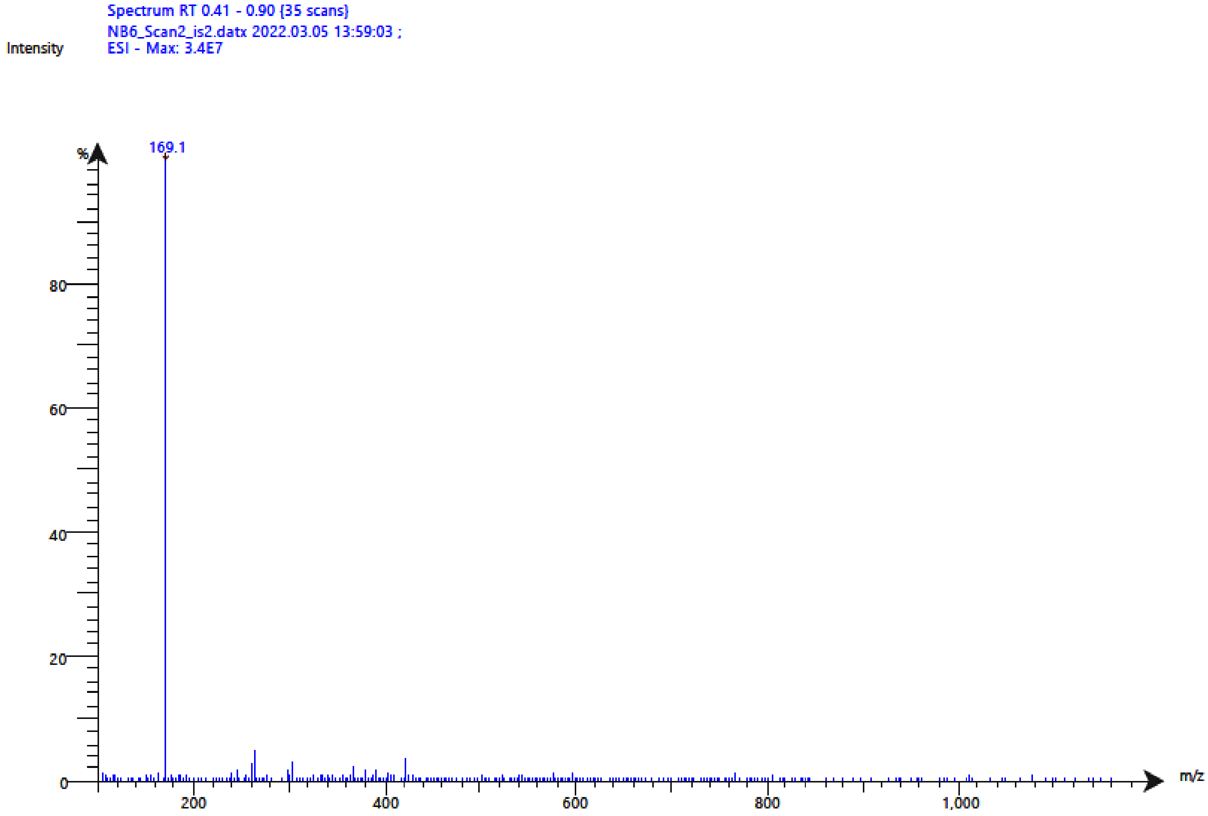

Figure S21. ESIMS "positive (A) and negative modes (B)" of compound (**5**)

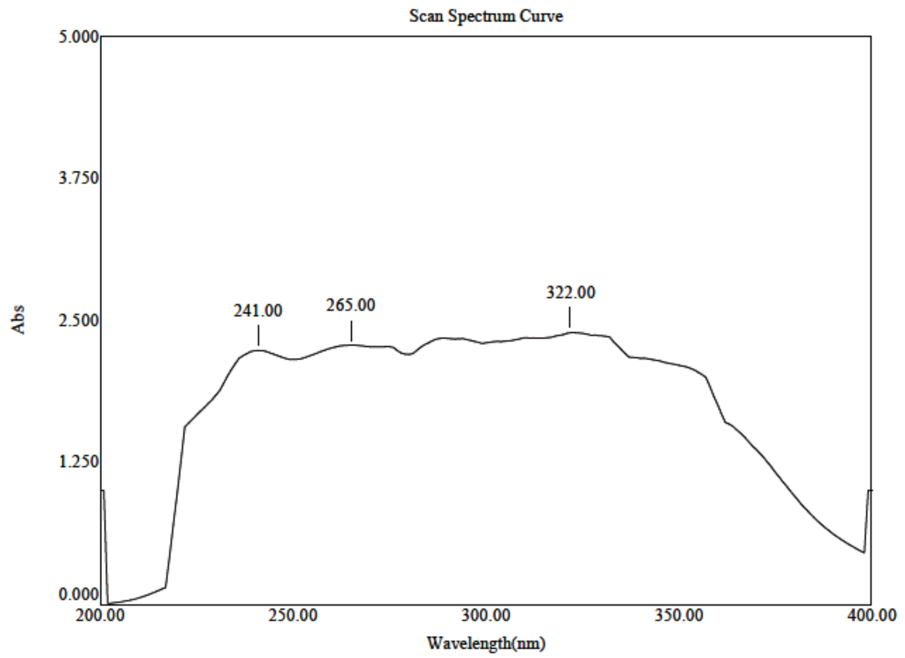

Figure S22. UV spectrum of compound (**6**) in MeOH

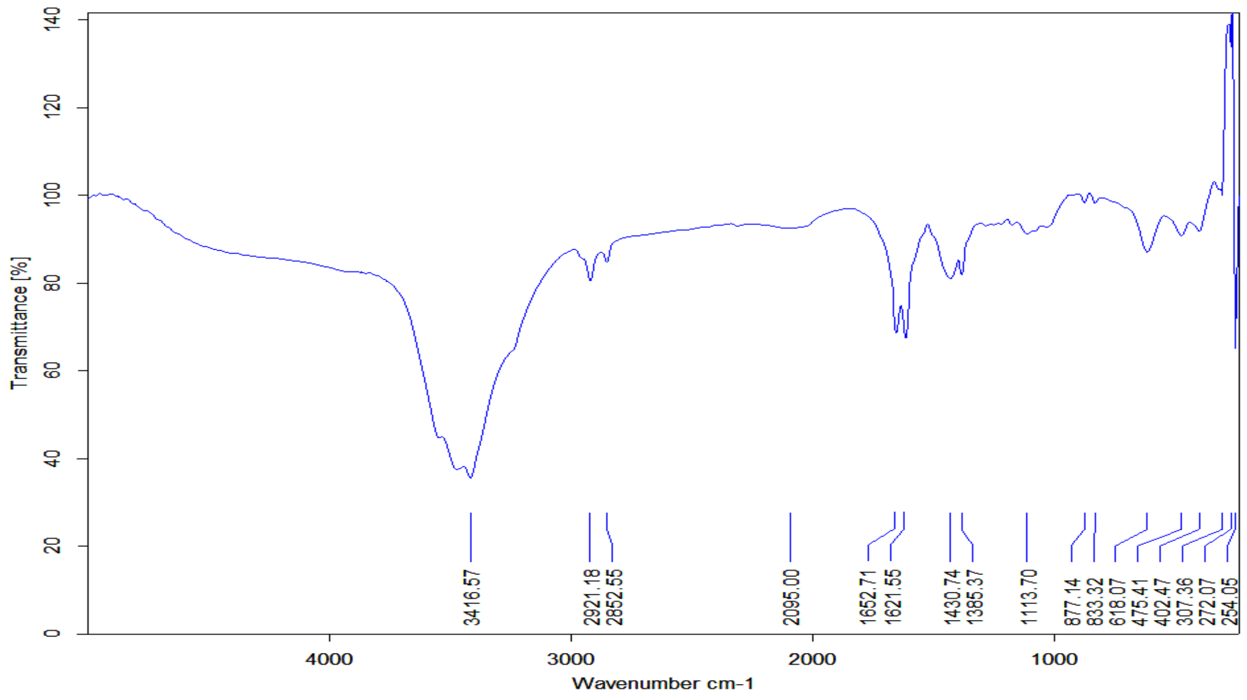

Figure S23. IR spectrum of compound (**6**) in KBr disc

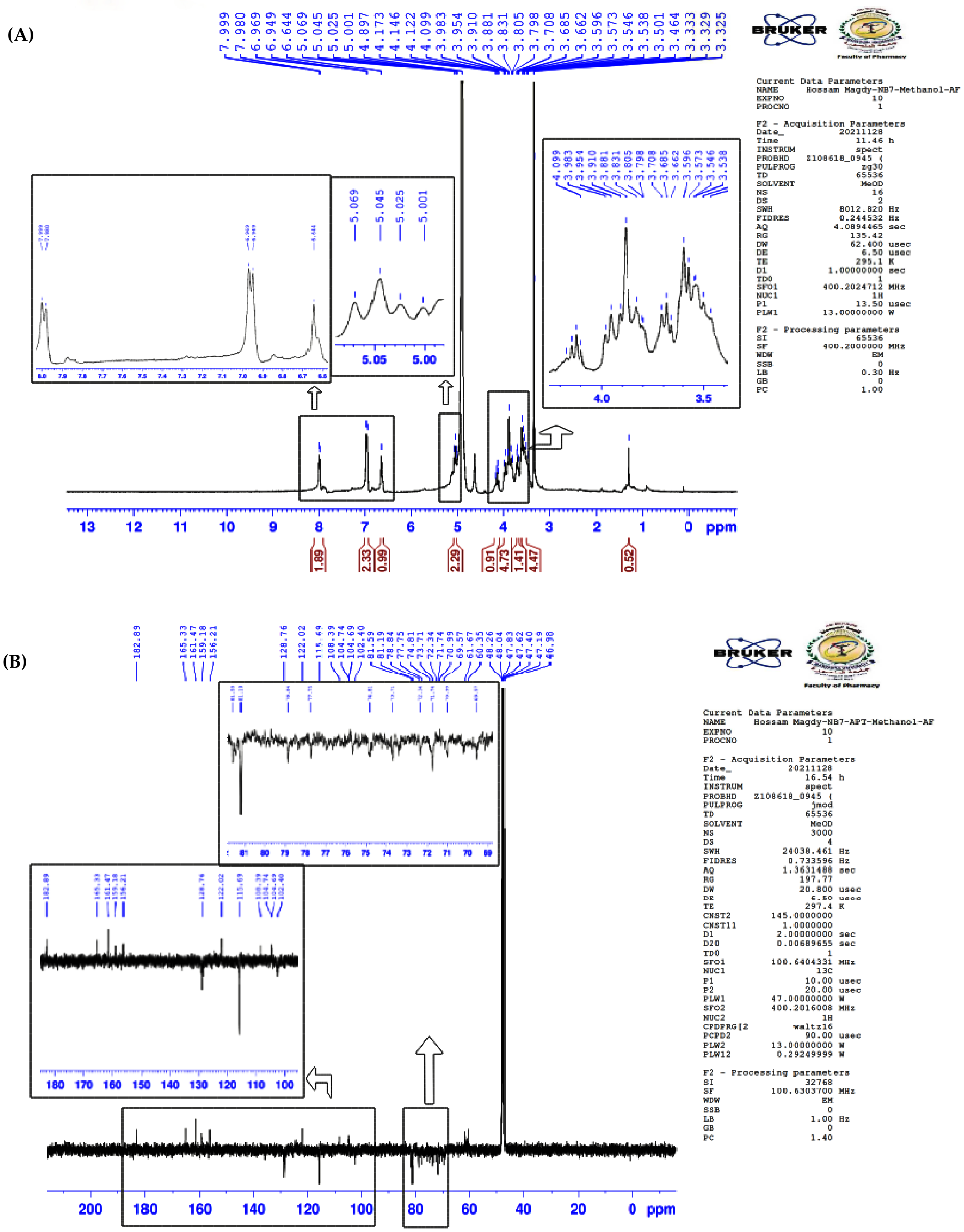

Figure S24. (A) ^{1}H and (B) APT NMR spectrum of compound (**6**) (CD_3OD)

(A)

(B)

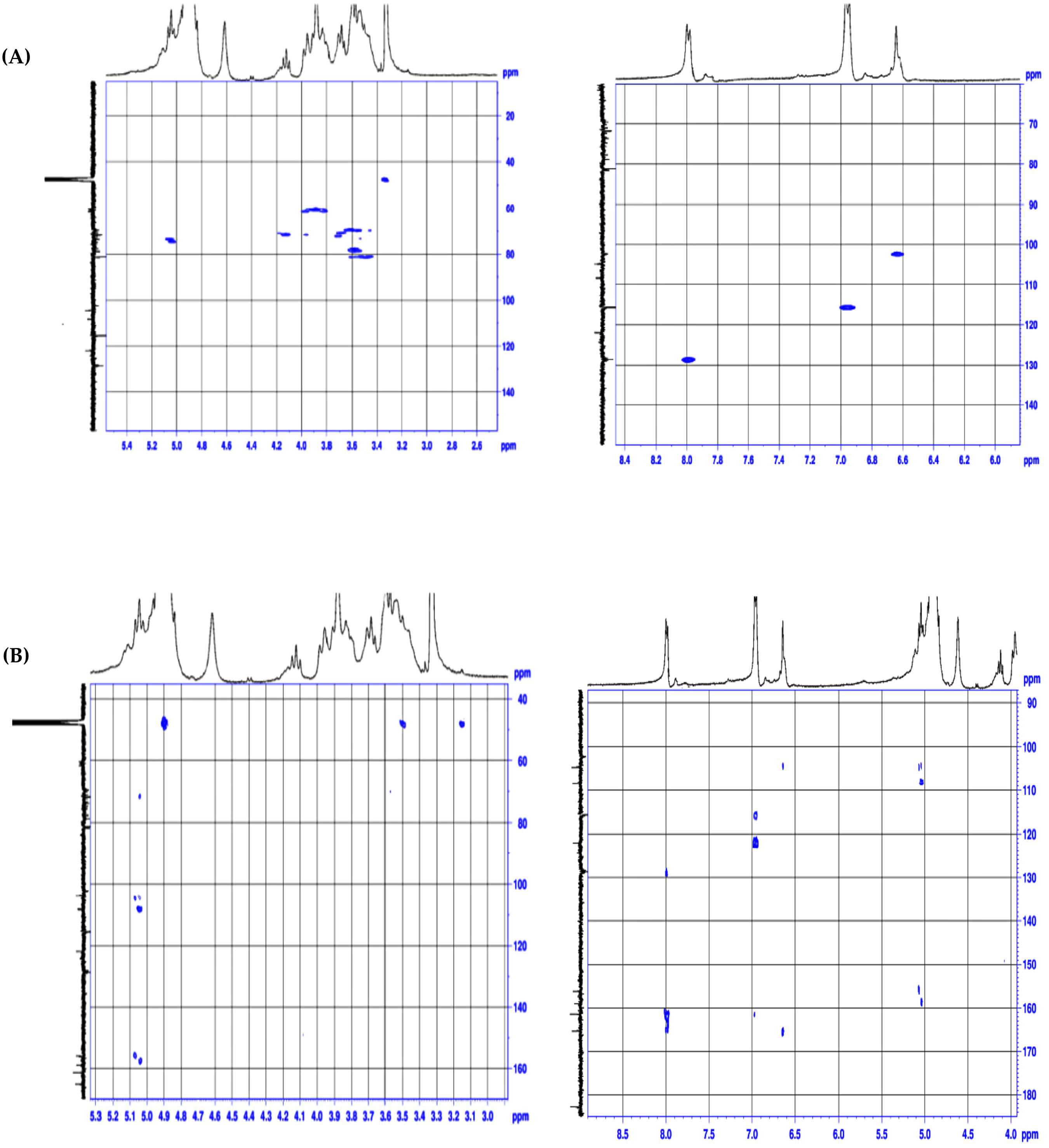

Figure S25. (A) HSQC and (B) HMBC spectrums of compound (**6**) (CD_3OD)

(A)

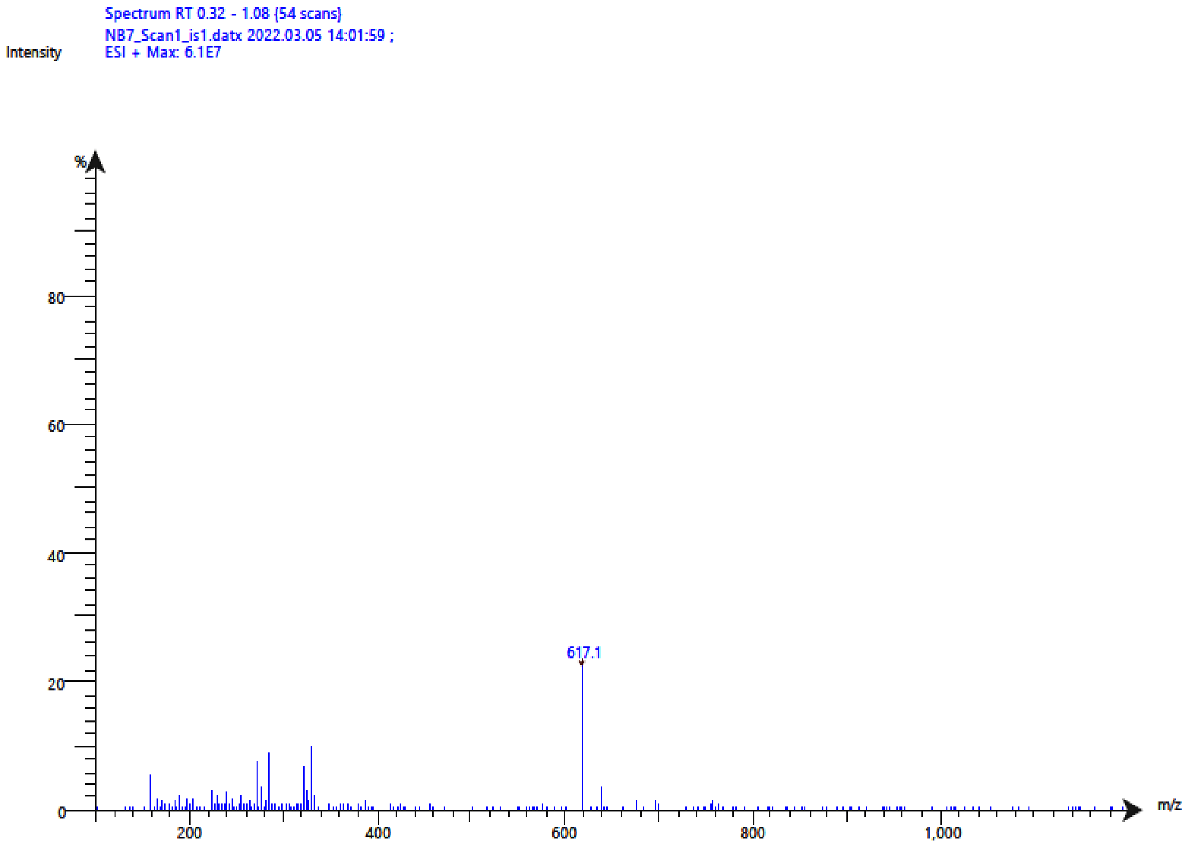

(B)

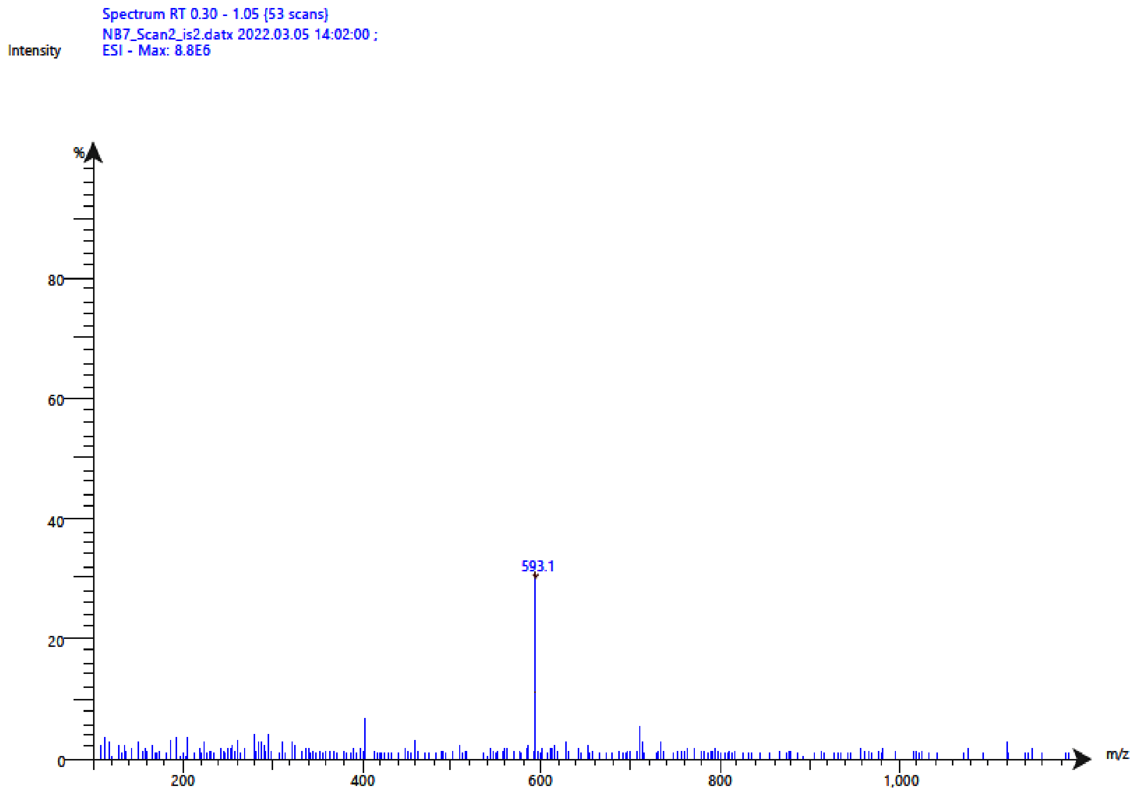

Figure S26. ESIMS "positive (A) and negative modes (B)" of compound (**6**)

(A) **(B)**

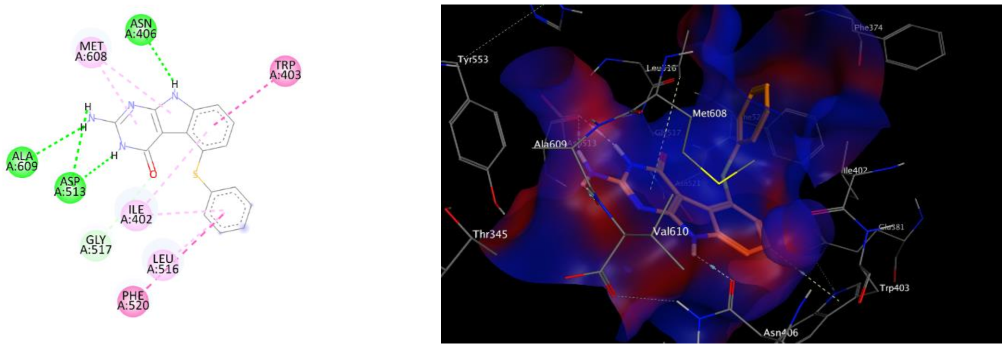

Figure S27. 2D (A) and 3D (B) patterns demonstrating the binding interaction of **1UE** into active site of TS-DHFR (PDB code: 4KY4). (A) Dotted pink and violet lines indicate hydrophobic interaction, and dotted green lines indicate hydrogen bonds. (B) Blue color surface refers a hydrophobic area and red color surface refers hydrophilic area.

(A) **(B)**

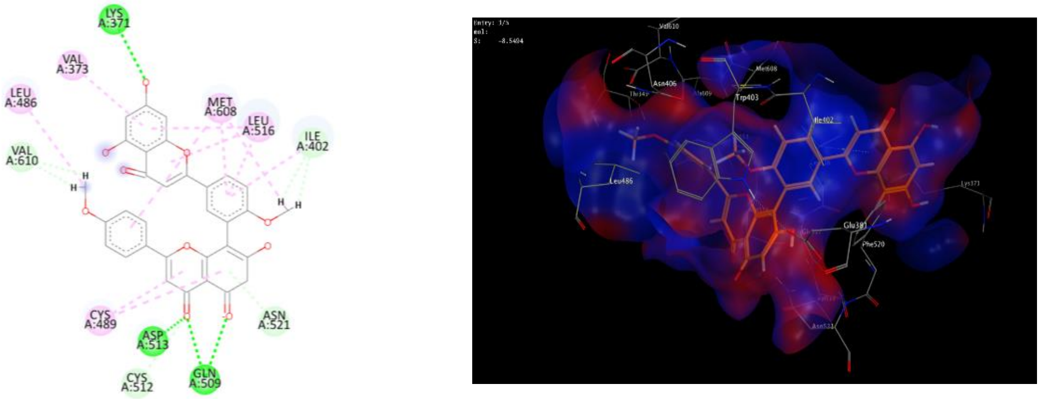

Figure S28. 2D (A) and 3D (B) patterns demonstrating the binding interaction of **Compound (1)** into active site of TS-DHFR (PDB code: 4KY4). (A) Dotted pink lines indicate hydrophobic interaction, and dotted green lines indicate hydrogen bonds. (B) Blue color surface refers a hydrophobic area and red color surface refers hydrophilic area.

(A) **(B)**

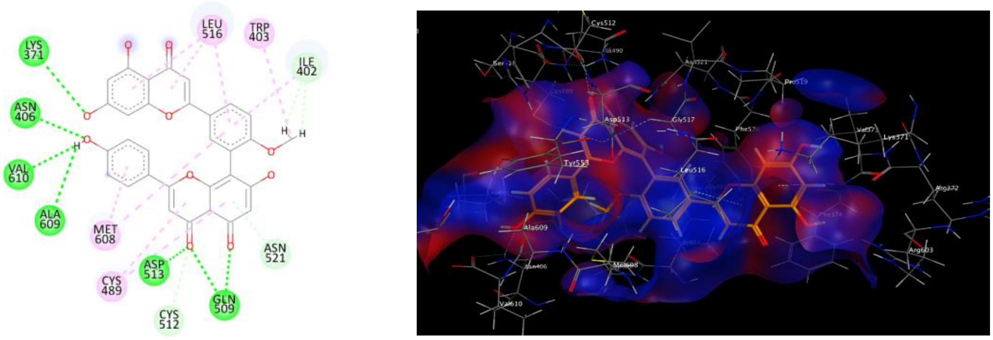

Figure S29. 2D (A) and 3D (B) patterns demonstrating the binding interaction of **Compound (2)** into active site of TS-DHFR (PDB code: 4KY4). (A) Dotted pink lines indicate hydrophobic interaction, and dotted green lines indicate hydrogen bonds. (B) Blue color surface refers a hydrophobic area and red color surface refers hydrophilic area.

(A)

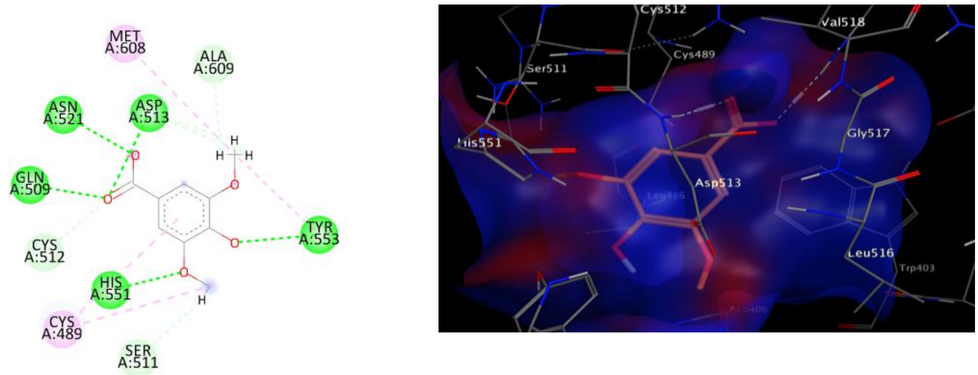

Figure S30. 2D (A) and 3D (B) patterns demonstrating the binding interaction of **Compound (3)** into active site of TS-DHFR (PDB code: 4KY4). (A) Dotted pink lines indicate hydrophobic interaction, and dotted green lines indicate hydrogen bonds. (B) Blue color surface refers a hydrophobic area and red color surface refers hydrophilic area.

(A) **(B)**

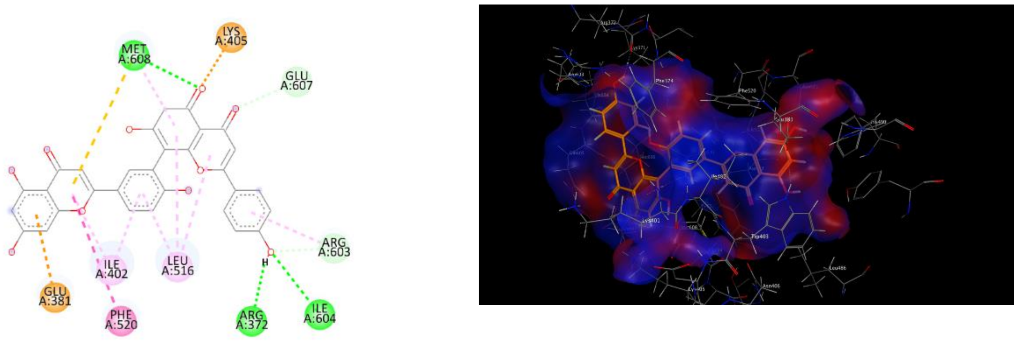

Figure S31. 2D (A) and 3D (B) patterns demonstrating the binding interaction of **Compound (4)** into active site of TS-DHFR (PDB code: 4KY4). (A) Dotted pink, violet and orange lines indicate hydrophobic interaction, and dotted green lines indicate hydrogen bonds. (B) Blue color surface refers a hydrophobic area and red color surface refers hydrophilic area.

(A) **(B)**

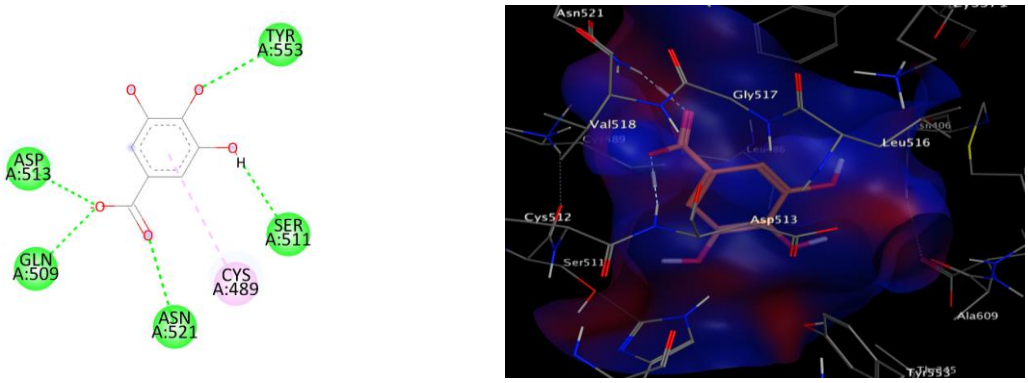

Figure S32. 2D (A) and 3D (B) patterns demonstrating the binding interaction of **Compound (5)** into active site of TS-DHFR (PDB code: 4KY4). (A) Dotted pink lines indicate hydrophobic interaction, and dotted green lines indicate hydrogen bonds. (B) Blue color surface refers a hydrophobic area and red color surface refers hydrophilic area.

(A) (B)

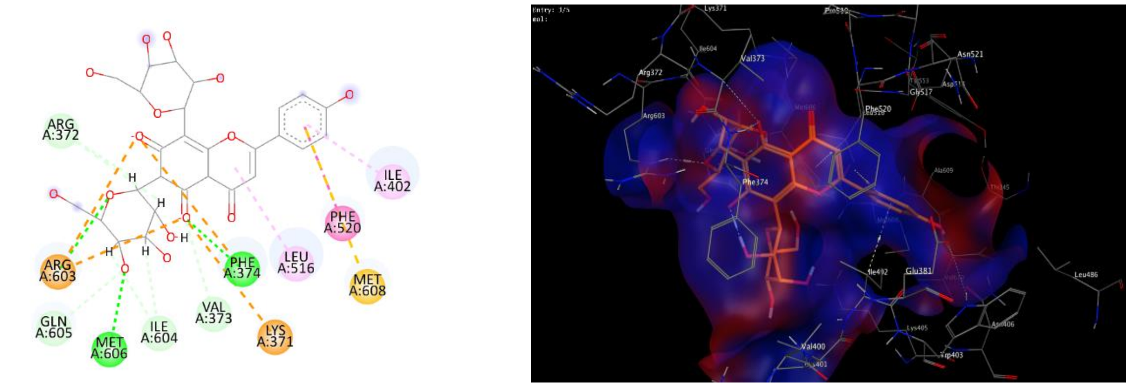

Figure S33. 2D (A) and 3D (B) patterns demonstrating the binding interaction of **Compound (6)** into active site of TS-DHFR (PDB code: 4KY4). (A) Dotted pink, violet and orange lines indicate hydrophobic interaction, and dotted green lines indicate hydrogen bonds. (B) Blue color surface refers a hydrophobic area and red color surface refers hydrophilic area.

(A) (B)

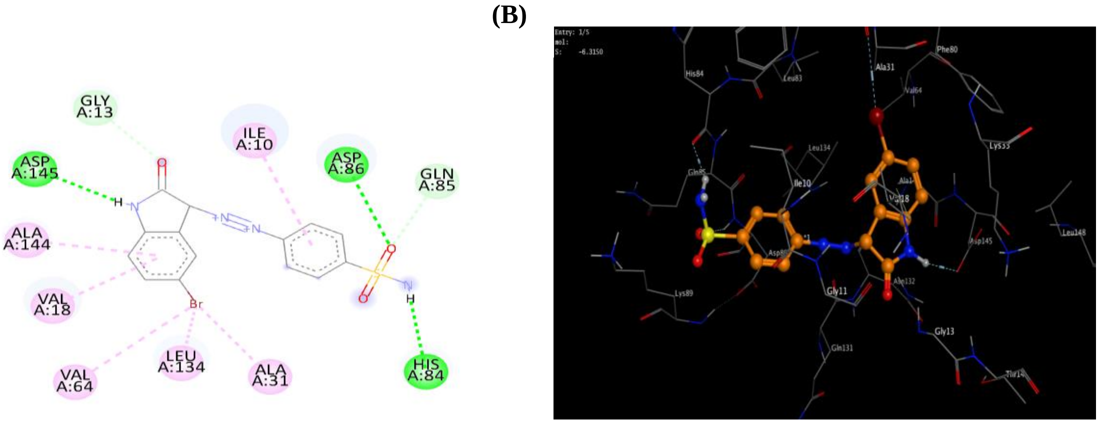

Figure S34. 2D (A) and 3D (B) patterns demonstrating the binding interaction of **(106)** into active site of CDK-2 (PDB ID: 1FVT). (A) Dotted pink lines indicate hydrophobic interaction, and dotted green lines indicate hydrogen bonds. (B) Blue color surface refers a hydrophobic area and red color surface refers hydrophilic area.

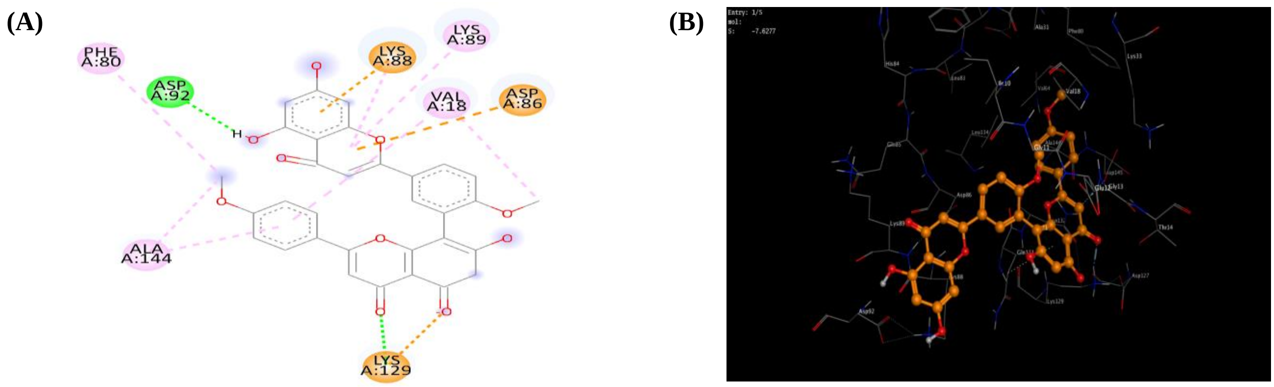

Figure S35. 2D (A) and 3D (B) patterns demonstrating the binding interaction of **compound (1)** into active site of CDK-2 (PDB ID: 1FVT). (A) Dotted pink and orange lines indicate hydrophobic interaction, and dotted green lines indicate hydrogen bonds. (B) Blue color surface refers a hydrophobic area and red color surface refers hydrophilic area.

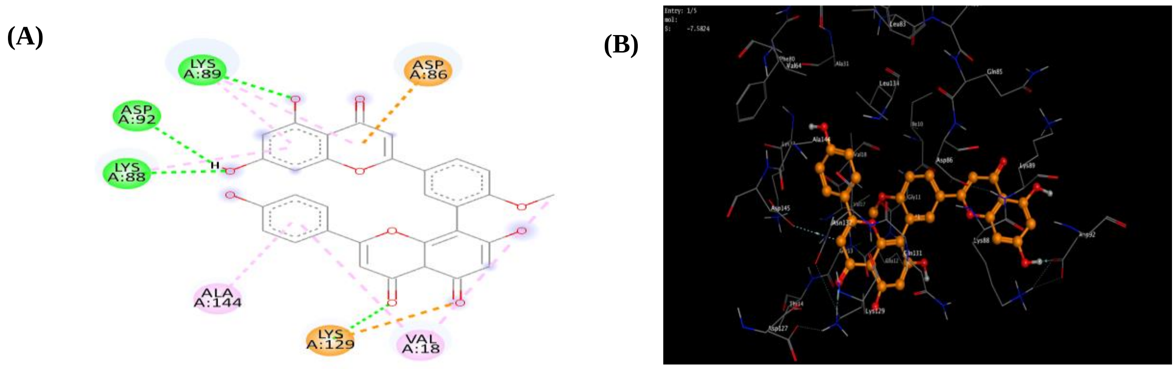

Figure S36. 2D (A) and 3D (B) patterns demonstrating the binding interaction of **compound (2)** into active site of CDK-2 (PDB ID: 1FVT). (A) Dotted pink, violet and orange lines indicate hydrophobic interaction, and dotted green lines indicate hydrogen bonds. (B) Blue color surface refers a hydrophobic area and red color surface refers hydrophilic area.

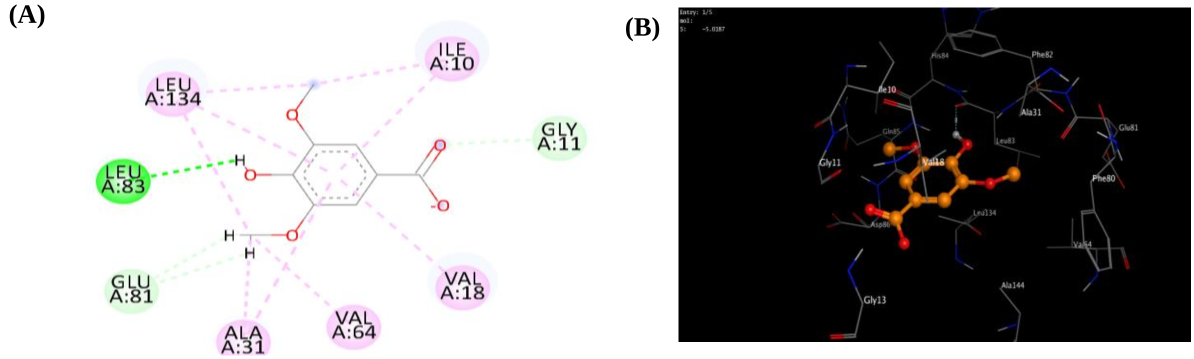

Figure S37. 2D (A) and 3D (B) patterns demonstrating the binding interaction of **compound (3)** into active site of CDK-2 (PDB ID: 1FVT). (A) Dotted pink lines indicate hydrophobic interaction, and dotted green lines indicate hydrogen bonds. (B) Blue color surface refers a hydrophobic area and red color surface refers hydrophilic area.

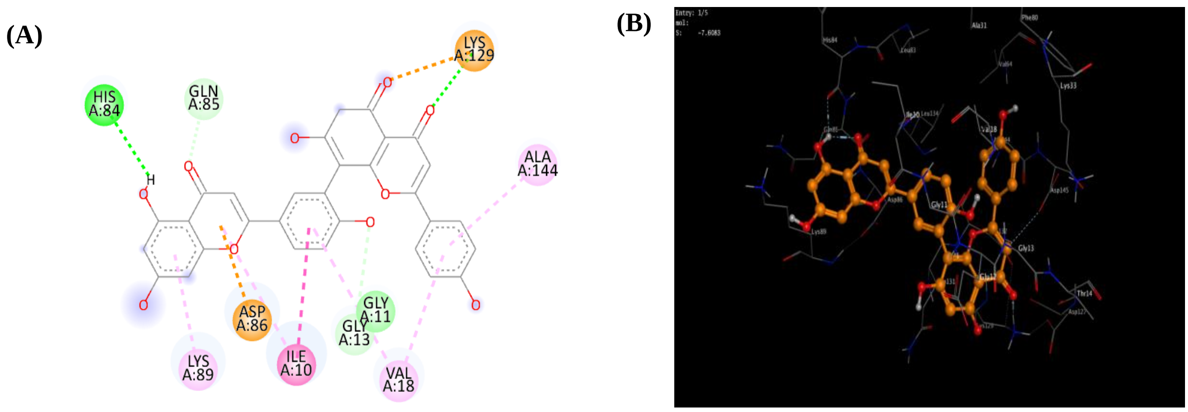

Figure S38. 2D (A) and 3D (B) patterns demonstrating the binding interaction of **compound (4)** into active site of CDK-2 (PDB ID: 1FVT). (A) Dotted pink, violet and orange lines indicate hydrophobic interaction, and dotted green lines indicate hydrogen bonds. (B) Blue color surface refers a hydrophobic area and red color surface refers hydrophilic area.

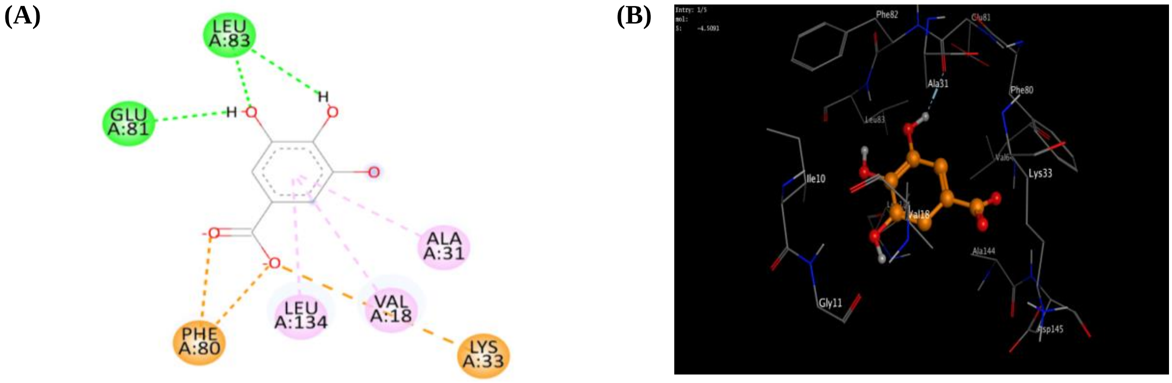

Figure S39. 2D (A) and 3D (B) patterns demonstrating the binding interaction of **compound (5)** into active site of CDK-2 (PDB ID: 1FVT). (A) Dotted pink and orange lines indicate hydrophobic interaction, and dotted green lines indicate hydrogen bonds. (B) Blue color surface refers a hydrophobic area and red color surface refers hydrophilic area.

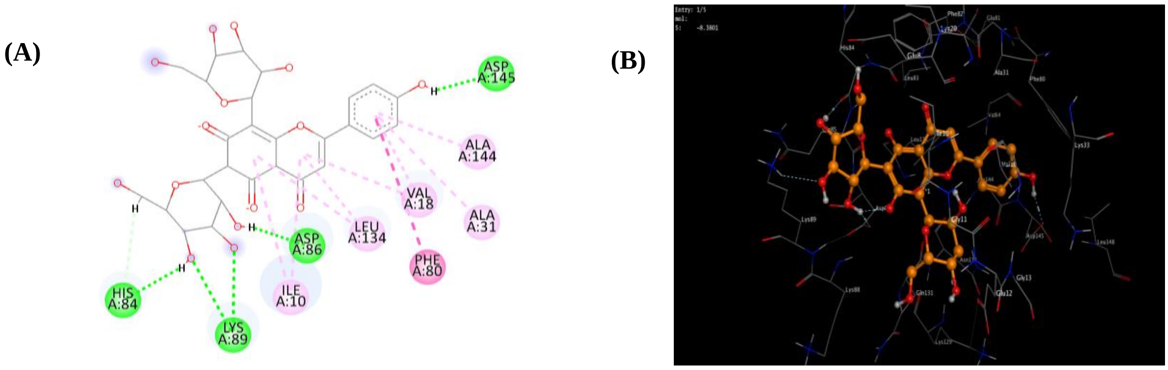

Figure S40. 2D (A) and 3D (B) patterns demonstrating the binding interaction of **compound (6)** into active site of CDK-2 (PDB ID: 1FVT). (A) Dotted pink and violet lines indicate hydrophobic interaction, and dotted green lines indicate hydrogen bonds. (B) Blue color surface refers a hydrophobic area and red color surface refers hydrophilic area.
